# Osteology of *Pseudochampsa ischigualastensis* gen. et comb. nov. (Archosauriformes: Proterochampsidae) from the Early Late Triassic Ischigualasto Formation of Northwestern Argentina

**DOI:** 10.1371/journal.pone.0111388

**Published:** 2014-11-26

**Authors:** M. Jimena Trotteyn, Martín D. Ezcurra

**Affiliations:** 1 CONICET, Consejo Nacional de Investigaciones Científicas y Técnicas, Ciudad Autónoma de Buenos Aires, Buenos Aires, Argentina; 2 INGEO, Instituto de Geología, Facultad de Ciencias Exactas, Físicas y Naturales, Universidad Nacional de San Juan, San Juan, San Juan, Argentina; 3 School of Geography, Earth and Environmental Sciences, University of Birmingham, Birmingham, West Midlands, United Kingdom; University of Pennsylvania, United States of America

## Abstract

Proterochampsids are crocodile-like, probably semi-aquatic, quadrupedal archosauriforms characterized by an elongated and dorsoventrally low skull. The group is endemic from the Middle-Late Triassic of South America. The most recently erected proterochampsid species is “*Chanaresuchus ischigualastensis*”, based on a single, fairly complete skeleton from the early Late Triassic Ischigualasto Formation of northwestern Argentina. We describe here in detail the non-braincase cranial and postcranial anatomy of this species and revisit its taxonomy and phylogenetic relationships. The phylogenetic analysis recovered ‘*Chanaresuchus ischigualastensis*’ as part of a trichotomy together with *Gualosuchus reigi* and *Chanaresuchus bonapartei*. Accordingly, “*Chanaresuchus ischigualastensis*” can be potentially more closely related to *Gualosuchus reigi*, or even *Rhadinosuchus gracilis*, than to *Chanaresuchus bonapartei*. In addition, after discussing previously claimed synapomorphies of *Chanaresuchus*, we could not find unambiguous support for the monophyly of the genus. As a result, we propose here the erection of the new genus *Pseudochampsa* for ‘*Chanaresuchus ischigualastensis*’, which results in the new combination *Pseudochampsa ischigualastensis*. The information provided here about the anatomy and taxonomy of *Pseudochampsa ischiguaslastensis* will be useful for future quantitative analyses focused on the biogeography and macroevolutionary history of proterochampsids.

## Introduction

The Triassic witnessed the origin, radiation and complete extinction of several diapsid clades, including morphological disparate forms such as kuehneosaurids, rhynchosaurs, erythrosuchids, doswelliids, proterochampsids, aetosaurs, and silesaurids among others [1]–[7]. These groups achieved generally a global distribution, but proterochampsids are endemic forms of the Middle and Late Triassic of South America [8]. Proterochampsids are crocodile-like, probably semi-aquatic, quadrupedal forms characterized by an elongated and in some cases a strongly dorsoventrally compressed skull [6]. There are eight nominal species described from the Ischigualasto-Villa Unión Basin of northwestern Argentina and the Paraná Basin of southern Brazil. The group is particularly important to understand the evolutionary radiation of Archosauriformes because has been interpreted as the sister-taxon of the crown-group Archosauria. A detail review of the overall anatomy, phylogeny and palaeobiology of the group was recently conducted by Trotteyn et al. [6] and we refer authors to that publication for a deeper overview of the clade.

The most recently erected proterochampsid species is ‘*Chanaresuchus ischigualastensis*’ from the early Late Triassic Ischigualasto Formation of northwestern Argentina. The species is known from a single, fairly complete skeleton (PVSJ 567) that was described preliminarily by Trotteyn et al. [9]. Subsequently, a detailed description of the braincase was published by Trotteyn & Haro [10]. In this contribution we redescribe in detail the holotype of ‘*Chanaresuchus ischigualastensis*’ and include this species for the first time in a quantitative phylogenetic analysis. The result of the analysis and discussion of previously claimed synapomorphies of *Chanaresuchus* do not support the monophyly of the genus and, as a result, we erect the new genus *Pseudochampsa* and the new combination *Pseudochampsa ischigualastensis*. A more complete knowledge of the anatomy and phylogenetic relationships of *Pseudochampsa ischigualastensis* will shed light on the evolution of proterochampsids and archosauriforms before the divergence of Archosauria.

## Materials and Methods

The type specimen of *Pseudochampsa ischigualastensis* (PVSJ 567) was studied in its repository with the explicit permission of the curator R. Martínez (Museo de Ciencias Naturales, Universidad Nacional de San Juan) (see Acknowledgements). Information about the discovery of the specimen was provided by V. Contreras (INGEO, Universidad Nacional de San Juan).

### Phylogenetic analysis

In order to test the phylogenetic relationships of *Pseudochampsa ischigualastensis* we included this species in the most recent and comprehensive data matrix focused on proterochampsids published so far, Dilkes & Arcucci [23]. In addition, we added ten characters to the original data set, resulting in a new data matrix composed of 16 taxa and 110 characters (Appendixes S1, S2). A recent preliminary quantitative phylogenetic analysis found *Rhadinosuchus gracilis* as more closely related to *Chanaresuchus bonapartei* than to other proterochampsids [24]. The former species will not be included here because is part of an independent study that will be published elsewhere. The quantitative phylogenetic analysis was conducted under equally-weighted parsimony using TNT 1.1 [25] using the implicit enumeration algorithm. Character 21 was treated as additive (ordered) following Dilkes & Arcucci [23]. Zero length branches were collapsed following the search (rule 1 of Coddington and Scharff [26]). As measures of tree support, decay indices ( = Bremer supports) were calculated and a bootstrap resampling analysis, with 10,000 pseudoreplicates, was performed reporting both absolute and GC (i.e. difference between the frequency that the original group and the most frequent contradictory group are recovered in the pseudoreplicates) frequencies.

### Nomenclatural Acts

The electronic edition of this article conforms to the requirements of the amended International Code of Zoological Nomenclature, and hence the new names contained herein are available under that Code from the electronic edition of this article. This published work and the nomenclatural acts it contains have been registered in ZooBank, the online registration system for the ICZN. The ZooBank LSIDs (Life Science Identifiers) can be resolved and the associated information viewed through any standard web browser by appending the LSID to the prefix “http://zoobank.org/”. The LSID for this publication is: urn:lsid:zoobank.org:pub:FD3F7D8A-D1FE-42E8-BC8A-5739D1188371. The electronic edition of this work was published in a journal with an ISSN, and has been archived and is available from the following digital repositories: PubMed Central and LOCKSS.

### Institutional abbreviations

BSPG, Bayerische Staatssammlung für Paläontologie und Geologie, Munich, Germany; CA, Colégio Anchieta, Porto Alegre, Brazil; MCP, Museo de Ciencias e Tecnologia, Porto Alegre, Brazil; MCZ, Museum of Comparative Zoology, Harvard University, Boston, USA; NM, National Museum, Bloemfontein, South Africa; PIN, Paleontological Institute of the Russian Academy of Sciences, Moscow, Russia; PULR, Paleontología, Universidad Nacional de La Rioja, La Rioja, Argentina; PVL, Paleontología de Vertebrados, Instituto “Miguel Lillo”, San Miguel de Tucumán, Argentina; PVSJ, División de Paleontologia de Vertebrados del Museo de Ciencias Naturales y Universidad Nacional de San Juan, San Juan, Argentina; RC, Rubidge Collection, Wellwood, Graaff-Reinet, South Africa; SAM-PK, Iziko South African Museum, South Africa; SMNS, Staatliches Museum für Naturkunde, Stuttgart, Germany; USNM, National Museum of Natural History (formerly United States National Museum), Smithsonian Institution, Washington DC, USA.

## Systematic Palaeontology

DIAPSIDA Osborn, 1903 [11] sensu Laurin (1991) [12]

ARCHOSAUROMORPHA Huene, 1946 [13] sensu Dilkes (1998) [14]

ARCHOSAURIFORMES Gauthier et al., 1988 [15] sensu Gauthier et al. (1988) [15]

PROTEROCHAMPSIA Bonaparte, 1971 [16] sensu Kischlat (2000) [17]

PROTEROCHAMPSIDAE Sill, 1967 [18] sensu Trotteyn (2011) [19]

*Pseudochampsa* gen. nov.

urn:lsid:zoobank.org:act: 8FA98C77-CFF5-446C-9C99-C3F6F2CB1E10

### Etymology

The generic name (‘false crocodile’) is derived from the Greek words *pseudo* (false) and *champsa* (crocodile) in allusion to the crocodile-like body morphology of the animal.

### Diagnosis

Same as for its type species, *Pseudochampsa ischigualastensis*.

*Pseudochampsa ischigualastensis* (Trotteyn et al. 2012) [9] comb. nov.

Figures 1–13

**Figure 1:**
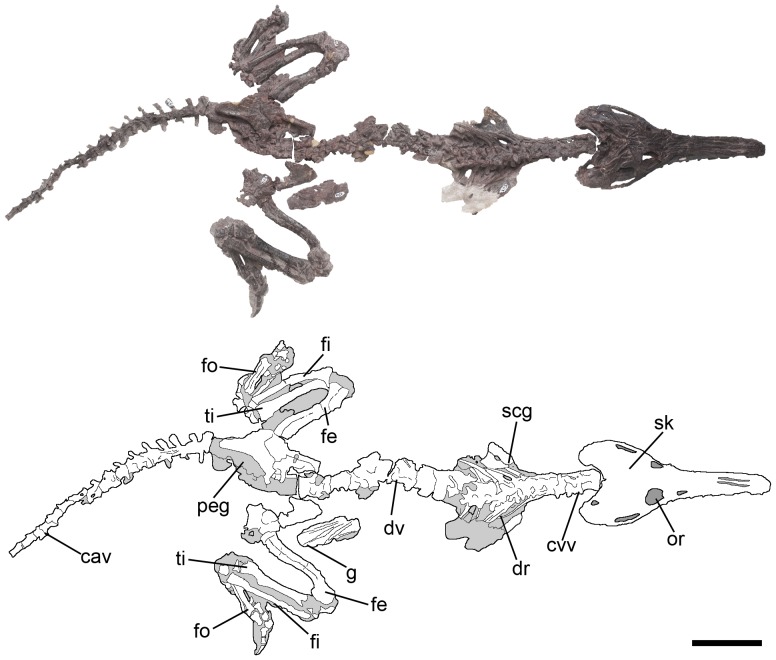
Holotype of *Pseudochampsa ischigualastensis* (PVSJ 567) as it was found in the field. Abbreviations: cav, caudal vertebrae; cvv, cervical vertebrae; dr, dorsal ribs; dv, dorsal vertebrae; fe, femur; fi, fibula; fo, foot; g, gastralia; or, orbit; peg, pelvic girdle; scg, scapular girdle; sk, skull; ti, tibia. Scale bar equals 5 cm.

**Figure 2:**
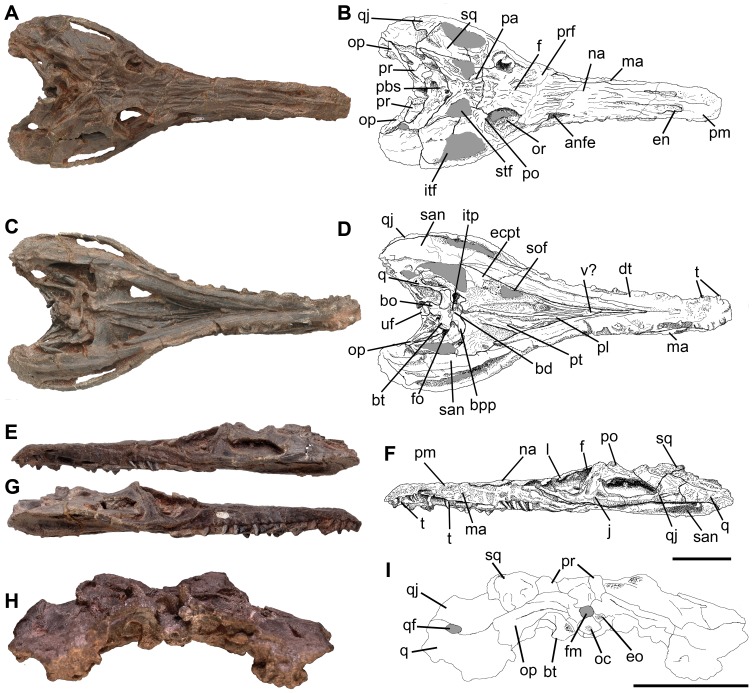
Skull of *Pseudochampsa ischigualastensis* (PVSJ 567) (A, B) in dorsal, (C, D) ventral, (E, F) left lateral, (G) right lateral, and (H, I) occipital views. Abbreviations: anfe, antorbital fenestra; bo, basioccipital; bpp, basipterygoid process; bd, basisphenoidal depression; bt, basal tubera; dt, dentary; ecpt, ectopterygoid; eo, exoccipital; en, external naris; f, frontal; fm, foramen magnum; fo, fenestra ovalis; itf, infratemporal fenestra; itp, intertuberal plate; j, jugal; l, lacrimal; ma, maxilla; na, nasal; oc, occipital condyle; op, opisthotic; or, orbit; pa, parietal; pbsp, parabasisphenoid; pl, palatine; pm, premaxilla; po, postorbital; pr, prootic; prf, prefrontal; pt, pterygoid; q, quadrate; qf, quadrate foramen; qj, quadratojugal; san, surangular; sof, suborbital fenestra; stf, supratemporal fenestra; sq, squamosal; t, tooth; uf, indeterminate bone fragment; v?, vomer?. Scale bars equal 5 cm.

**Figure 3:**
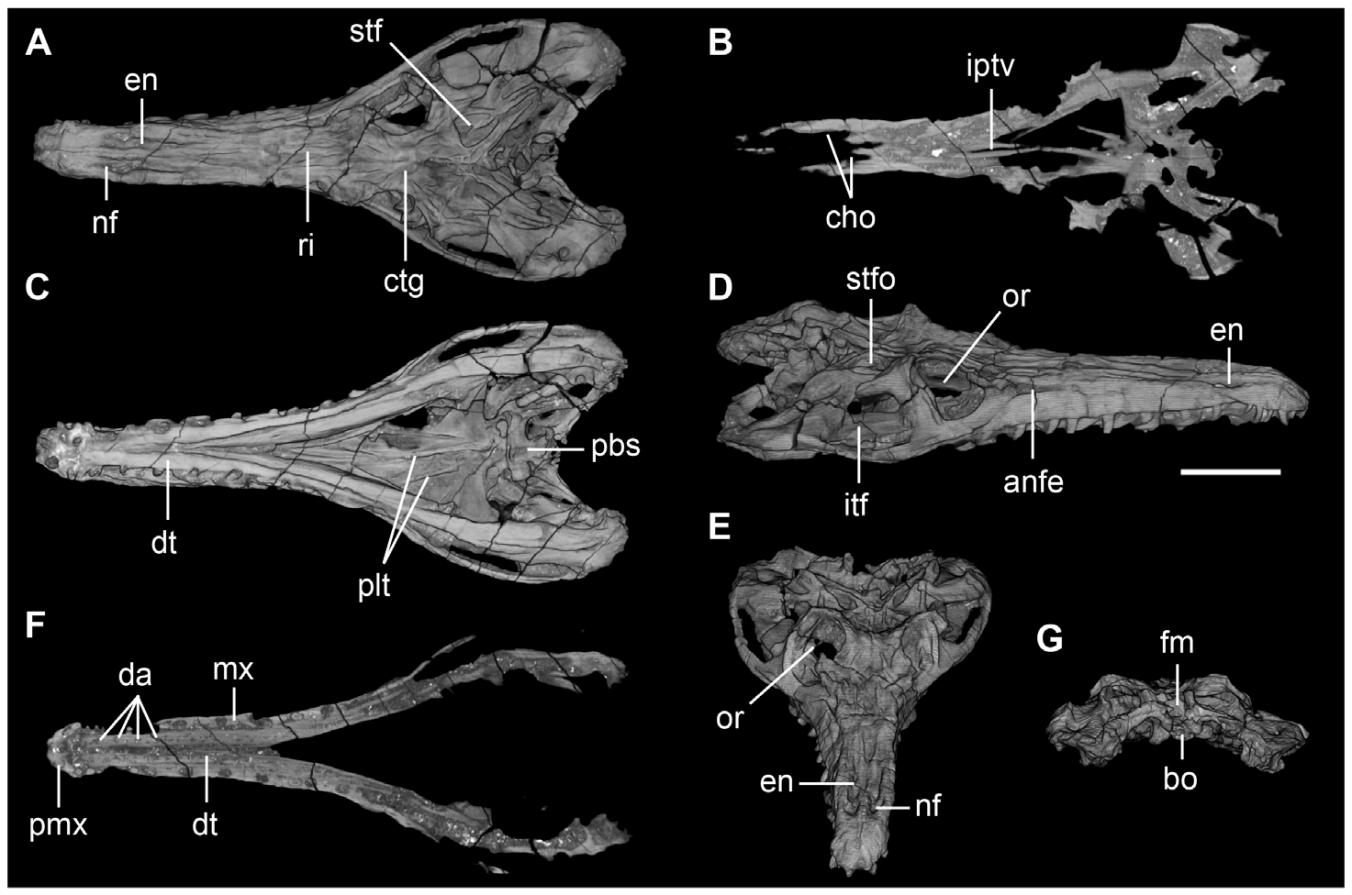
CT-scan slices and 3D reconstruction of the skull of *Pseudochampsa ischigualastensis* (PVSJ 567) in (A) dorsal view, (B) slice at level of the palate, (C) ventral, (D) dorsolateral, (E) and anterodorsal view, (F) slice at level of the dentary alveoli, and (G) occipital view. Abbreviations: anfe, antorbital fenestra; bo, basioccipital; cho, choanae; ctg, centre of growth; da, dentary alveoli; dt, dentary; en, external naris; iptv, interpterygoid vacuity; itf, infratemporal fenestra; fm, foramen magnum; mx, maxilla; nf, narial fossa; or, orbit; pbs, parabasisphenoid; pmx, premaxilla; plt, palate teeth; ri, ridge; stf, supratemporal fenestra, stfo, supratemporal fossa. Scale bar equals 5 cm.

**Figure 4:**
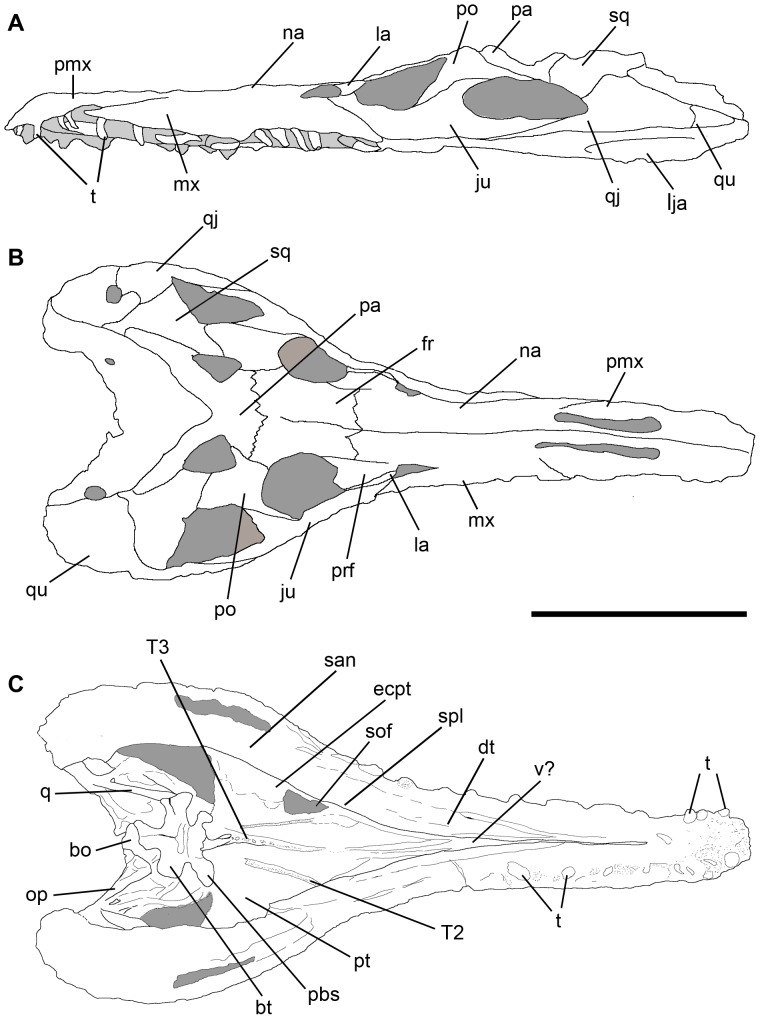
Line drawings showing sutures in the skull of *Pseudochampsa ischigualastensis* (PVSJ 567) in (A) left lateral, (B) dorsal, and (C) ventral views. Abbreviations: bo, basioccipital; bt, basal tubera; dt, dentary; ecpt, ectopterygoid; fr, frontal; ju, jugal; la, lacrimal; lja, lower jaw; mx, maxilla; na, nasal; op, opisthotic; pa, parietal; pbs, parabasisphenoid; pmx, premaxilla; po, postorbital; prf, prefrontal; pt, pterygoid; qj, quadratojugal; q, quadrate; san, surangular; sof, suborbital fenestra; sq, squamosal; spl, splenial; t, teeth; T2, palate tooth row 2; T3, palate tooth row 3; v?, vomer?. Scale bar equals 5 cm.

**Figure 5:**
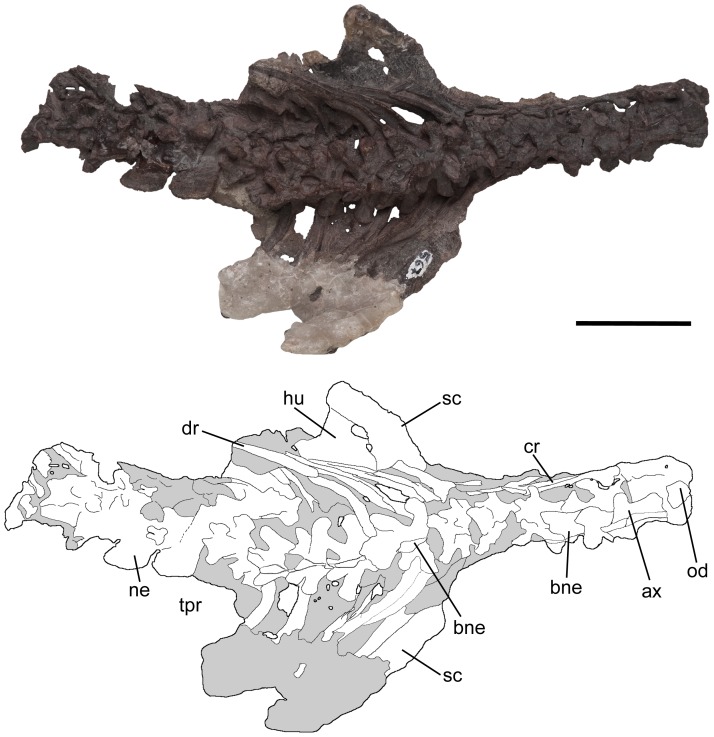
Cervical and dorsal vertebrae, and scapulacoracoids of *Pseudochampsa ischigualastensis* (PVSJ 567) in dorsal view. Abbreviations: ax, axis; bne, base of neural spine; cr, cervical rib; dr, dorsal rib; hu, humerus; ne¸neural spine; od, odontoid; sc, scapulacoracoid; tpr, transverse process. Scale bar equals 5 cm.

**Figure 6:**
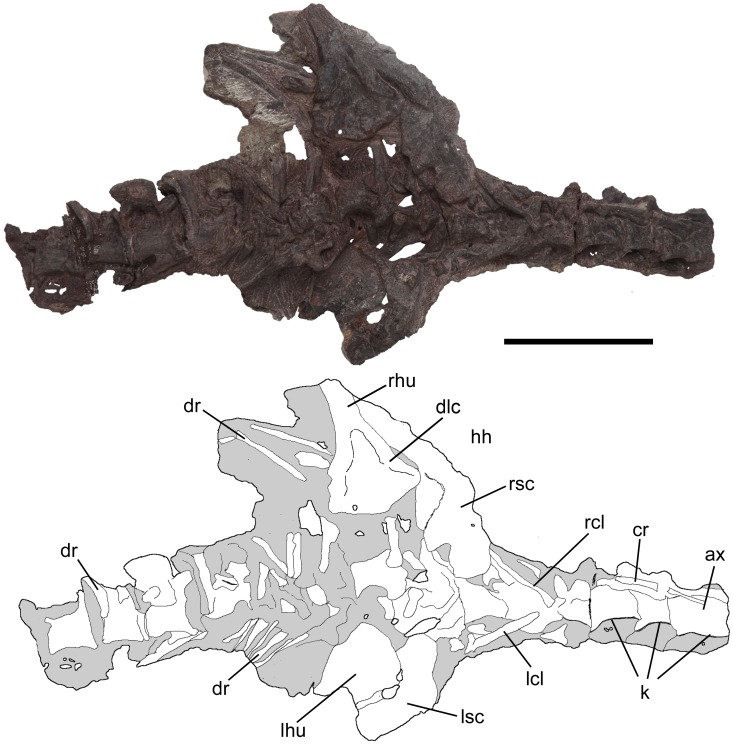
Cervical and dorsal vertebrae, and scapulacoracoids of *Pseudochampsa ischigualastensis* (PVSJ 567) in ventral view. Abbreviations: ax, axis; cr, cervical ribs; dlc, deltopectoral crest; dr, dorsal ribs; hh, humeral head; k, ventral keel; lcl, left clavicle; lhu, left humerus; lsc, left scapulacoracoid; rcl, right clavicle; rhu, right humerus; rsc, right scapulacoracoid. Scale bar equals 5 cm.

**Figure 7:**
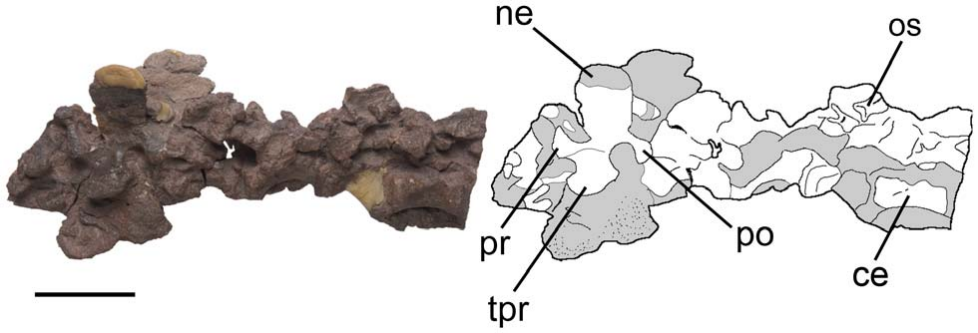
Posterior dorsal vertebrae of *Pseudochampsa ischigualastensis* (PVSJ 567) in dorsolateral view. Abbreviations: ce, centrum; ne, neural spine; os, osteoderm; po, postzygapophysis; pr, prezygapophysis; tpr, transverse process. Scale bar equals 3 cm.

**Figure 8:**
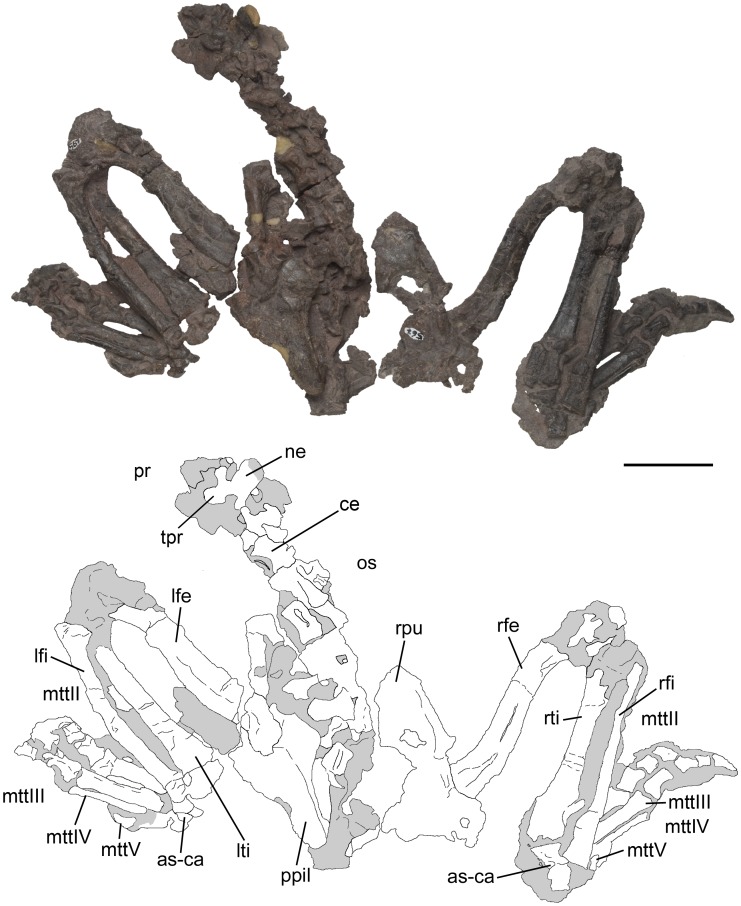
Pelvic girdle and hindlimbs of *Pseudochampsa ischigualastensis* (PVSJ 567) in dorsal view. Abbreviations: as-ca, astragalus-calcaneum; ce, centrum; mttII–V, metatarsals II–V; lfe, left femur; lfi, left fibula; lti, left tibia; ne, neural spine; os, osteoderm; ppil, postacetabular process of ilium; pr, prezygapophysis; rfe, right femur; rfi, right fibula; rpu, right pubis; rti, right tibia; tp, transverse process. Scale bar equals 5 cm.

**Figure 9:**
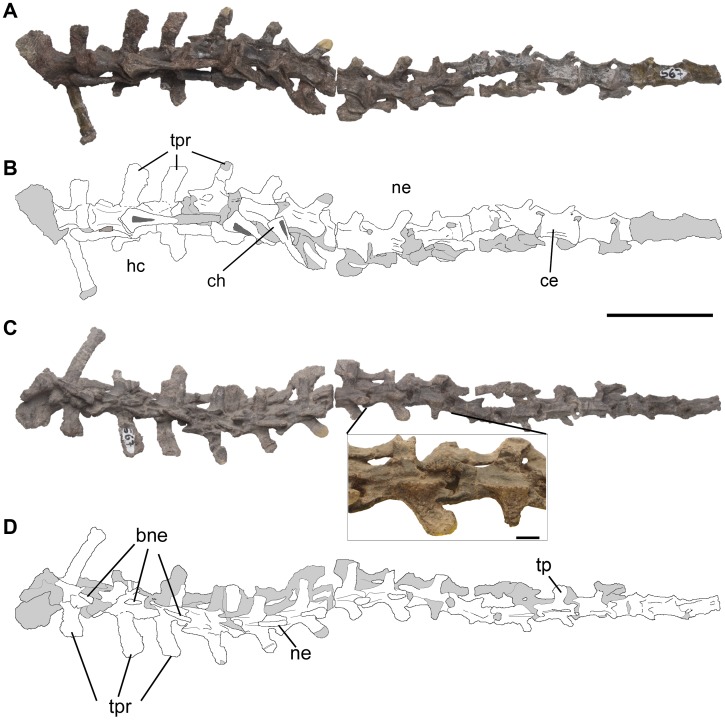
Caudal vertebrae of *Pseudochampsa ischigualastensis* (PVSJ 567) in (A, B) ventral/left lateral, and (C, D) dorsal/right lateral views. Close-up of two middle caudal vertebrae in dorsal view in (C). Abbreviations: bne, base of neural spine; ce, centrum; ch, chevron; hc, hemal canal; ne, neural spine; tpr, transverse process. Scale bar equals 5 cm and 1 cm in close-up of (C).

**Figure 10:**
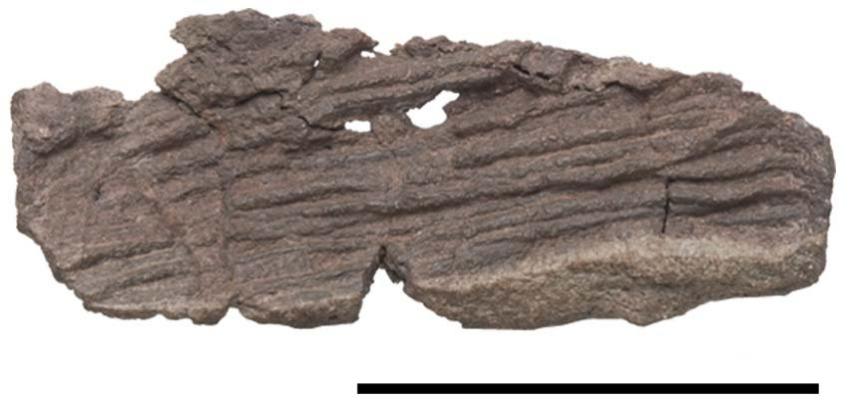
Gastralia of *Pseudochampsa ischigualastensis* (PVSJ 567). Scale bar equals 5 cm.

**Figure 11:**
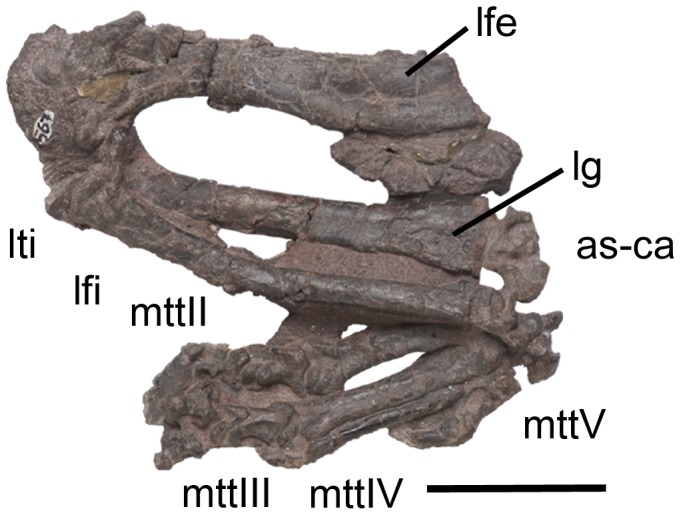
Left hindlimb of *Chanaresuchus ischigualastensis* (PVSJ 567) in anterior view. Abbreviations: as-ca, astragalus-calcaneum; lfi, left fibula; lfe, left femur; lg, lateral groove; lti, left tibia; mttII–V, metatarsals II–V. Scale bar equals 5 cm.

**Figure 12:**
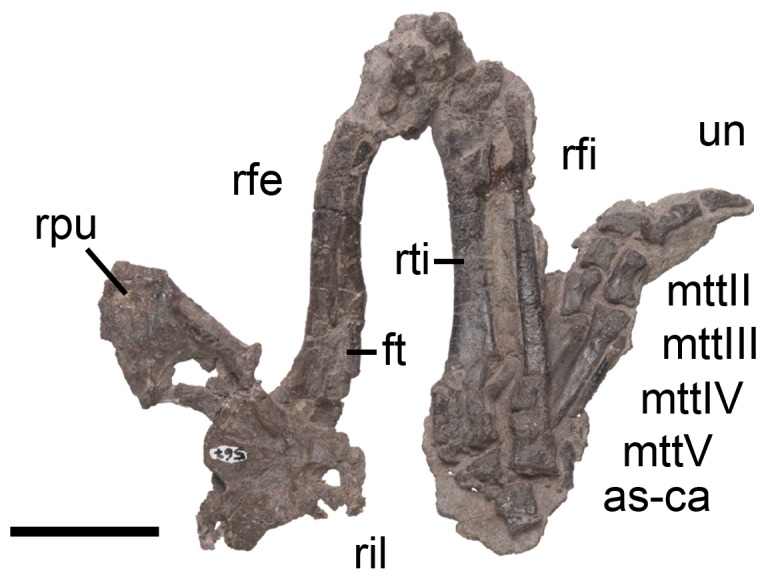
Right hindlimb of *Pseudochampsa ischigualastensis* (PVSJ 567) in anterior view. Abbreviations: as-ca, astragalus-calcaneum; ft, fourth trochanter; mttII–V, metatarsals II–V; rfi, right fibula; rfe, right femur; ril, right ilium; rti, right tibia; un, ungueal. Scale bar equals 5 cm.

**Figure 13:**
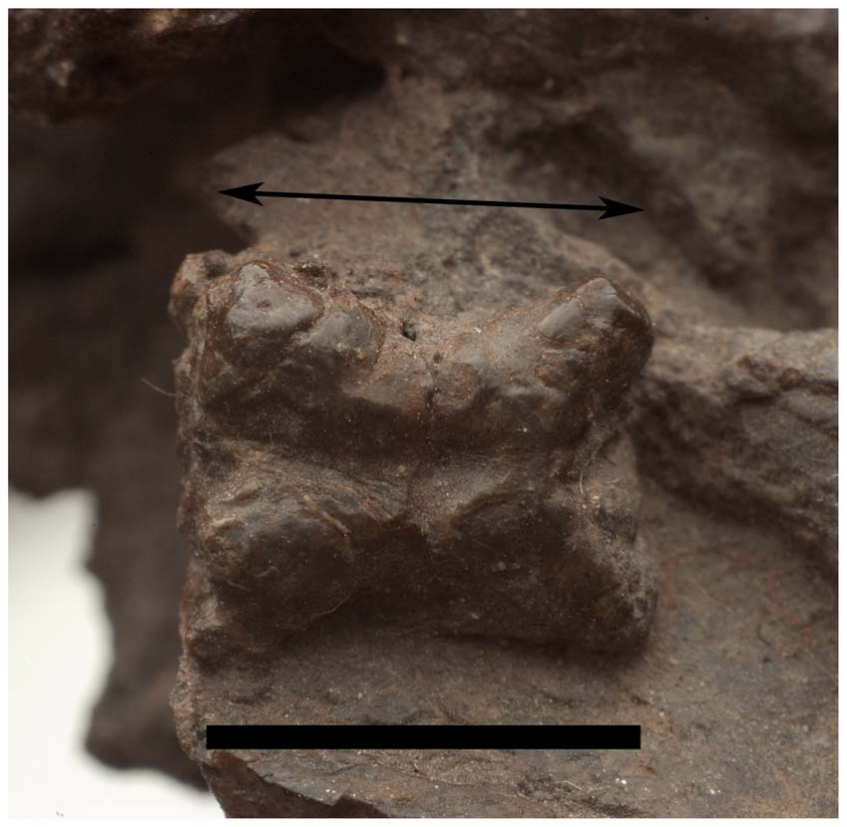
Osteoderm of *Pseudochampsa ischigualastensis* (PVSJ 567) in dorsal view. The black arrow indicates anteroposterior orientation. Scale bar equals 1 cm.

2012 *Chanaresuchus ischigualastensis*, Trotteyn et al., p. 485, 486, 488, figs. 2–4.

### Holotype

PVSJ 567, fairly complete, articulated skeleton including skull with fully occluded lower jaws, complete vertebral series lacking the distal half of the tail, several cervical and dorsal ribs, some haemal arches, some gastralia, pectoral girdle, both partial humeri, partial pelvic girdle, both femora, tibiae, fibulae, tarsals, and pes (Fig. 1).

### Emended diagnosis

*Pseudochampsa ischigualastensis* is distinguished from other proterochampsids, including *Chanaresuchus bonapartei*, on the basis of the following unique combination of character-states: basicranium transversely broad (basal tubera width/parabasisphenoidal complex axial length ratio = 0.31) and with transversely oriented basal tubera; paroccipital processes with dorsoventrally expanded distal end; lower jaws without retroarticular process; caudal vertebrae with a median longitudinal groove on the ventral surface of the centrum, and pre- and postzygapophyses strongly divergent from the median line; astragalus lacking foramina on the posterior groove; and osteoderms with an ornamentation consisting only of a longitudinal groove (modified from Trotteyn et al. [9]).

### Type Horizon and Locality

Cancha de Bochas Member of the Ischigualasto Formation (late Carnian–earliest Norian [20]), Ischigualasto-Villa Union Basin [21], [22], Ischigualasto Provincial Park, San Juan Province, northwestern Argentina.

### Nomenclatural comment

Proterochampsia is phylogenetically defined as the most recent common ancestor of *Proterochampsa* and all its descendants, but not *Crocodylus* and/or *Vultur* (stem-based, Kischlat [17]). Proterochampsidae is defined as the least inclusive group that is composed of *Chanaresuchus bonapartei* and *Proterochampsa barrionuevoi* but not *Euparkeria capensis*, *Passer domesticus* nor *Crocodylus niloticus* (node-based, Trotteyn [19]). Proterochampsia and Proterochampsidae possess the same taxonomic content under the phylogenetic topology recovered here (see below), but both taxa differ in that one is defined as a stem-based clade and the other as a node-based clade. Therefore, both taxa are valid and used in the Systematic Palaeontology section.

## Description

The skull of *Pseudochampsa ischigualastensis* is fairly complete, but taphonomically dorsoventrally compressed (Fig. 2). The skull length represents approximately 0.8 times the length of the presacral vertebral series (Fig. 1), resembling the condition in *Tropidosuchus romeri* (PVL 4601). By contrast, the skull is proportionally larger with respect to its postcranium in *Proterochampsa barrionuevoi* [27]. The neck of *Pseudochampsa ischigualastensis* is proportionally short, representing approximately half of the length of the dorsal series. The distal half of the tail is not preserved, but it is at least as long as the dorsal series. The forelimbs are represented only by the humeri and, as a result, its proportion with respect to the rest of the skeleton cannot be determined. The hindlimb is relatively long, being approximately as long as the entire dorsal series.

### Skull

The overall cranial morphology of *Pseudochampsa ischigualastensis* resembles that of extant crocodiles, with a dorsoventrally low skull (but lower than it should have been in life due to taphonomic artefacts) and elongated snout (Figs. 2–4), as it was previously highlighted for other proterochampsids [6]. The orbits are dorsally facing as a result of the strong dorsoventral compression of the skull, as occurs in *Proterochampsa barrionuevoi* (PVSJ 77), *Proterochampsa nodosa* (MCP 1694 PV), some specimens of *Chanaresuchus bonapartei* (PULR 07, PVL 4575) and *Doswellia kaltenbachi* (USNM 214823). The skull of *Pseudochampsa ischigualastensis* is sub-triangular in dorsal view, with a considerably transversely expanded postorbital region with respect to the snout. The external nares are tear-drop-shaped in dorsal view, with a tapering posterior end, and situated close to the median-line of the snout, as in other proterochampsids. The skull roof is strongly ornamented by ridges arranged in a radial pattern on the frontals and nasals (Fig. 2A, B, Fig. 3A), as also occurs in *Chanaresuchus bonapartei* (PULR 07, PVL 4575), *Gualosuchus reigi* (PULR 05) and *Rhadinosuchus gracilis* (BSPG AS XXV 50, 51). By contrast, the skulls of *Proterochampsa barrionuevoi* (PVSJ 77) and *Proterochampsa nodosa* (MCP 1694 PV) are ornamented by nodular tubercles on its lateral surface and longitudinal ridges on the nasals, and *Tropidosuchus romeri* possesses a pair of longitudinal ridges on the frontals and parietals (PVL 4601, 4602, 4606). The strong ornamentation of the skull roof of *Pseudochampsa ischigualastensis* obscures some sutures on the skull roof. The antorbital fenestra is dorsolaterally facing and has a relatively small size (Fig. 3D), as also occurs in *Chanaresuchus bonapartei* (PULR 07), *Proterochampsa barrionuevoi* (PVSJ 77) and *Proterochampsa nodosa* (MCP 1694 PV). The infratemporal fenestra opens dorsolaterally and is trapezoidal (Fig. 2A, B: stf; Fig. 3A: stf), resembling the condition in other proterochampsids (e.g. *Chanaresuchus bonapartei*: PULR 07, PVL 4575; *Proterochampsa barrionuevoi*: PVSJ 77; *Proterochampsa nodosa*: MCP 1694 PV). The supratemporal fenestra is relatively small and sub-triangular, contrasting with the suboval supratemporal fenestra of *Chanaresuchus bonapartei* (PULR 07, PVL 4575, 4586). The posttemporal fenestra is small and represented by a slit between the parietal and paraoccipital process in occipital view.

The palate of *Pseudochampsa ischigualastensis* is formed by the premaxillae, maxillae, vomers, palatines, pterygoids and ectopterygoids. The internal nares (choanae) are strongly anteroposteriorly elongated (Figs. 3B: cho), resembling the condition in other proterochampsids (e.g. *Chanaresuchus bonapartei*: PULR 07). The interpterygoid vacuity is strongly transversely reduced and visible anteriorly to the level of the suborbital fenestra. The suborbital fenestra is relatively small and subtriangular, with a transversely oriented posterior margin and an anterior apex.

#### Premaxilla

The suture between the premaxilla and maxilla is visible in lateral view (Figs. 2–4), but is obscured by strong ornamentation on the dorsal surface of the snout. The suture with the maxilla is diagonal, being anteroventrally-to-posterodorsally oriented. The premaxillary body is well anteroposteriorly elongated and slightly downturned, as occurs in other proterochampsids (e.g. *Proterochampsa barrionuevoi* [23]; *Chanaresuchus bonapartei*: PULR 07, PVL 4586; *Gualosuchus reigi*: PULR 05; *Rhadinosuchus gracilis*: BSPG AS XXV 50, 51). The lateral surface of the premaxilla is slightly anteroposteriorly convex and lacks ornamentation. The dorsal surface of the premaxilla possesses a shallow, poorly rimmed narial fossa, contrasting with the deeper narial fosa of some specimens of *Chanaresuchus bonapartei* (PULR 07, PVL 4586). The medial and ventral surfaces of both premaxillae are obscured by the occluded lower jaws (Fig. 3F). The premaxilla has six tooth positions. The tooth crowns are labiolingually compressed and lack ornamentation on the enamel (e.g. longitudinal ridges, wrinkles) and wear facets. The last two premaxillary crowns are the highest of the series and more anterior crowns are subequal.

#### Maxilla

The maxillae (Figs. 2–4) are approximately parallel with each other on the snout, but posteriorly they curved strongly laterally and, as a result, diverge strongly from each other on the orbital region of the skull. The latter condition is also present in *Chanaresuchus bonapartei* (PVL 4575) and *Gualosuchus reigi* (PVL 4576). By contrast, the maxillae of *Proterochampsa barrionuevoi* (PVSJ 77) and *Proterochampsa nodosa* (MCP 1694 PV) diverge posteriorly on the snout, from the premaxilla-maxilla suture. The maxilla of *Pseudochampsa ischigualastensis* possesses an extremely elongated anterior process, situated anteriorly to the antorbital fenestra. The anterior process of the maxilla should have had an extensive contact with the nasal, but this suture cannot be discerned in the preserved specimen. The transition between the anterior and ascending processes of the maxilla is gradual on the dorsal margin of the bone. There is a sharp, right-angled change in slope between the lateral and dorsal surfaces of the maxilla along these processes. As a result, the maxilla participates on the dorsal surface of the snout, as also occurs in *Chanaresuchus bonapartei* (PULR 07, PVL 4586), *Gualosuchus reigi* (PULR 05) and *Rhadinosuchus gracilis* (BSPG AS XXV 50, 51). The ascending process is low and extends posterodorsally between the antorbital fenestra and the nasal, forming the anterodorsal border of the antorbial fenestra. The horizontal process extends posteriorly below the antorbital fenestra and lacks an antorbital fossa, resembling the condition in *Proterochampsa barrionuevoi* [23], *Proterochampsa nodosa* (MCP 1694 PV), *Cerritosaurus binsfeldi* (CA w/n), *Tropidosuchus romeri* (PVL 4601, 4606), *Chanaresuchus bonapartei* (PULR 07, PVL 4586) and *Gualosuchus reigi* (PULR 05). By contrast, *Rhadinosuchus gracilis* possesses an antorbital fossa on the anterior half of the horizontal process of the maxilla (BSPG AS XXV 50, 51). The maxilla has an extensive, diagonal suture with the anterior process of the jugal. The maxilla extends posteriorly slightly beyond the level of the posterior border of the orbit. The maxilla is contacted medially by the palatine, forming the posterolateral border of the choana. The posterior end of the horizontal process of the maxilla seems to have contacted the ectopterygoid, as also occurs in *Chanaresuchus bonapartei* (PVL 4586). The CT-revealed the presence of at least fifteen tooth positions in the maxilla (Fig. 3F). The teeth are deeply implanted in circular alveoli. The tooth crowns are labiolingually compressed and without denticles or ornamentation on the enamel. The fully erupted maxillary teeth are apicobasally taller than premaxillary teeth.

#### Nasal

The nasal (Figs. 2–4) is the longest bone of the skull roof, being at least two times anteroposteriorly longer than the frontal. The suture between the nasal and prefrontal is very difficult to discern and cannot be determined anteriorly above the antorbital fenestra. The preserved portion of the suture between the latter two bones is longitudinally oriented. The suture between the nasal and frontal is transversely oriented and interdigitated (Fig. 4B), as also occurs in *Chanaresuchus bonapartei* (PULR 07), *Cerritosaurus binsfeldi* (CA w/n) and *Proterochampsa barrionuevoi* [23]. This suture is situated immediately anteriorly to the level of the anterior border of the orbit. The dorsal surface of the nasal is ornamented by a series of mainly longitudinally oriented, thick ridges that originate from a centre of growth, resembling the condition in *Chanaresuchus bonapartei* (PULR 07, PVL 4586), *Gualosuchus reigi* (PULR 05) and *Rhadinosuchus gracilis* (BSPG AS XXV 50, 51). The centre of growth is situated slightly anteriorly to the level of the anterior border of the antorbital fenestra and adjacent to the medial margin of each nasal. The ridges extend posteriorly onto the frontal.

#### Lacrimal

The coarse ornamentation of the skull roof and poor preservation prevent determining completely the overall shape of the lacrimal (Figs. 2–4). The dorsal margin of the lacrimal possesses an extensive, mainly anteroposteriorly oriented suture with the prefrontal. This suture is visible in dorsal view. The lacrimal forms the posterodorsal border of the antorbital fenestra and lacks an antorbital fossa on the ventral process. By contrast, *Chanaresuchus bonapartei* (PVL 4575, 4586), *Rhadinosuchus gracilis* (BSPG AS XXV 50, 51) and *Cerritosaurus binsfeldi* (CA w/n) possess an antorbital fossa along the entire anterior half of the ventral process of the lacrimal. The ventral process of the lacrimal is mainly dorsoventrally oriented, but with a low anterior component, resembling the condition in other proterochampsids (e.g. *Chanaresuchus bonapartei*: PVL 4575, 4586; *Gualosuchus reigi*: PVL 4576).

#### Jugal

The jugal (Figs. 2–4) is triradiate, with the base of the ascending process perpendicular to the bases of the anterior and posterior processes. The anterior process is anteroposteriorly very long and forms the entire ventral border of the orbit. This process possesses a dorsal kink at the point of maximum ventral development of the orbit. From this point, the anterior process tapers gradually anteriorly and its anterior end participates in the posteroventral border of the antorbital fenestra. The anterior end of the jugal possesses a short dorsal prong that contacts the lacrimal. The slender anterior process of *Pseudochampsa ischigualastensis* resembles that of *Chanaresuchus bonapartei* (PULR 07, PVL 4586), *Gualosuchus reigi* (PULR 05) and *Tropidosuchus romeri* (PVL 4601, PVL 4602), but differs from the more robust process of *Proterochampsa barrionuevoi* (PVSJ 77). There is a thick, longitudinal ridge extending along the lateral surface of the anterior and posterior processes of the jugal, resembling the condition in *Chanaresuchus bonapartei* (PULR 07, PVL 4575, 4586) and *Gualosuchus reigi* (PULR 05, PVL 4576). The ascending process is mainly vertical, but with a low posterior component. It forms the anteroventral border of the infratemporal fenestra, but is excluded from the posterior border of the orbit by the ventral process of the postorbital (Figs. 2E–G, 4A). The suture between the jugal and quadratojugal is diagonal, with the posterior process of the jugal extending dorsally to the anterior process of the quadratojugal, resembling the condition in other proterochampsids (e.g. *Gualosuchus reigi*: PVL 4576).

#### Prefrontal

The prefrontal (Figs. 2–4) of *Pseudochampsa ischigualastensis* is sub-triangular in dorsal view, with tapering anterior and posterior ends. The prefrontal is slightly laterally expanded in front of the orbit, resembling the condition in *Chanaresuchus bonapartei* (PULR 07) and *Gualosuchus reigi* (PULR 05). By contrast, the prefrontal is considerably more laterally expanded in *Tropidosuchus romeri* (PVL 4601, 4606). It is very difficult to determine if the prefrontal reached anteriorly the ascending process of the maxilla. The posterior process of the prefrontal forms the anterior half of the dorsal border of the orbit and contacts medially the frontal. The prefrontal seems to articulate with the lacrimal through an interdigitate suture.

#### Frontal

The frontals (Figs. 2–4) are not fused with each other. The pair of frontals is approximately as long as transversely wide, as also occurs in other proterochampsids (e.g. *Proterochampsa barrionuevoi* [23]). The lateral margin of the frontal possesses a long, anteroposteriorly concave notch that forms the dorsal border of the orbit in dorsal view. The orbital margin of the frontal is elevated dorsally, as also occurs in other proterochampsids (e.g. *Proterochampsa barrionuevoi* [23]; *Chanaresuchus ischigualastensis*: PULR 07). The posterolateral corner of the frontal articulates with the postorbital, in a posteromedially-to-anterolaterally oriented suture. The frontal-parietal suture is strongly interdigitated and mainly transversely oriented (Fig. 4B). As mentioned above, the dorsal surface of the frontal possesses a strong ornamentation (Fig. 2 A, B; Fig. 3A), but the crests are lower than in the nasal, as also occurs in *Chanaresuchus bonapartei* (PULR 07, PVL 4575). The best developed crests on the frontal are aligned to the sagittal axis of the skull and adjacent to the median line. The other crests are obliquely oriented to the sagittal axis of the skull and form together with the former crests a radial pattern of ornamentation.

#### Postfrontal

The postfrontal is absent in *Pseudochampsa ischigualastensis*, as occurs in other proterochampsids [6].

#### Postorbital

The postorbital (Figs. 2–4) is a triradiate bone that forms the posterodorsal border of the orbit, most of the dorsal border of the infratemporal fenestra, and the anterolateral border of the supramteporal fenestra. The ascending process of the postorbital is strongly anterodorsally oriented and almost straight. It contacts the parietal posteromedially and the frontal anteromedially through irregular sutures. A robust, well developed ridge is present adjacent to the orbital margin of the postorbital, being extended along the ascending and ventral processes of the bone, as also occurs in *Chanaresuchus bonapartei* (PULR 07) and *Proterochampsa barrionuevoi* (PVSJ 77). The ventral process of the postorbital is extended up to the ventral margin of the orbit, possessing an extensive diagonal suture with the ascending process of the jugal. The posterior process of the postorbital tapers posteriorly and forms most of the dorsal border of the infratemporal fenestra, contrasting with the condition in *Proterochampsa barrionuevoi* [23]. The suture with the anterior process of the squamosal is diagonal, being anterodorsally-to-posteroventreally oriented.

#### Squamosal

The squamosal (Figs. 2–4) is composed of an anterior process and a ventral one that form a wide angle with each other. The anterior process is relatively anteroposteriorly short and forms the posterior half of the lateral border of the supratemporal fenestra, whereas it is almost excluded from the dorsal border of the infratemporal fenestra by the posterior process of the postorbital. The squamosal receives the quadrate head, and the area of articulation between both bones is exposed in lateral and occipital views. The squamosal is not extended posteriorly beyond the level of the quadrate head. The squamosal participates in the posterolateral corner of the occipital crest, in a simple, anteromedially-to-posterolaterally oriented suture with the parietal. The ventral process is relatively long, anteroposteriorly broad and forms most of the posterior border of the infratemporal fenestra. By contrast, the ventral process of the squamosal forms only the dorsal half of the posterior border of the infratemporal fenestra in *Proterochampsa barrionuevoi* (PVSJ 77, [23]).

#### Quadratojugal

The quadratojugal (Figs. 2–4) is L-shaped in lateral view, with a dorsal and an anterior process forming a slightly acute angle with each other. The quadratojugal forms the posteroventral border of the infratemporal fenestra and possesses a distinct notch on the posteroventral corner of this opening, as occurs in other proterochampsids [23]. The anterior process of the quadratojugal contacts the jugal, forming a completely closed ventral border of the infratemporal fenestra and extending anteriorly close to the level of the base of the ascending process of the jugal (Fig. 2E–G). The quadratojugal possesses an extensive contact with the quadrate and forms the lateral border of a sub-circular and moderately large quadrate foramen.

#### Parietal

The suture between both parietals cannot be distinguished (Figs. 2–4). However, the presence of a potential fusion between both parietals at the median line of the skull roof cannot be determined because of the coarse ornamentation on this region. Both parietals are strongly constricted transversely by the medial apexes of the supratemporal fenestrae. The parietal has a transversely very narrow and deep supratemporal fossa, as also occurs in *Chanaresuchus bonapartei* (PULR 07, PVL 4586), *Tropidosuchus romeri* (PVL 4601, 4606), *Cerritosaurus binsfeldi* (CA w/n) and *Gualosuchus reigi* (PVL 4576). By contrast, *Proterochampsa barrionuevoi* lacks a supratemporal fossa [23]. Both parietals form a transversely wide separation between the supratemporal fenestrae, forming the entire medial and posterior borders of the opening. The posterolateral processes of the parietal diverge in an approximately right angle with each other, resembling the condition in *Chanaresuchus bonapartei* (PULR 07), *Tropidosuchus romeri* (PVL 4601, 4606), *Cerritosaurus binsfeldi* (CA w/n) and *Gualosuchus reigi* (PVL 4576). By contrast, in *Proterochampsa barrionuevoi* (PVSJ 77) and *Proterochampsa nodosa* (MCP 1694 PV) both posterolateral processes diverge from each other in an obtuse angle in dorsal view. The parietal contacts posterolaterally the squamosal and posteriorly the supraoccipital and exoccipital-opisthotic. A pair of parallel, longitudinal ridges extends on the dorsal surface of the parietals, as occurs in *Chanaresuchus bonapartei* (PULR 07, PVL 4586), *Tropidosuchus romeri* (PVL 4601, 4606), *Cerritosaurus binsfeldi* (CA w/n) and *Gualosuchus reigi* (PVL 4576). From these ridges extend subsidiary ridges diagonally and transversely, forming a radial ornamentation pattern, resembling the condition in *Chanaresuchus bonapartei* (PULR 07, PVL 4586) and *Gualosuchus reigi* (PVL 4576). By contrast, the latter ridges are not present in *Cerritosaurus binsfeldi* (CA w/n), *Tropidosuchus romeri* (PVL 4601, 4606) and *Gualosuchus reigi* (PVL 4576). A pair of anteromedially-to-posterolaterally oriented ridges extends onto the proximal half of the dorsal surface of the posterolateral process of the parietal.

#### Quadrate

The quadrate (Figs. 2–4) possesses an extensive, mainly dorsoventrally oriented suture with the quadratojugal, and forms the dorsal, ventral and medial borders of the quadrate foramen. The posterior surface of the proximal end of the quadrate possesses a weak contact with the paroccipital process. The quadrate head articulates on the ventral surface of the squamosal. The pterygoid wing of the quadrate has a considerable ventromedial development, contacting the pterygoid anteriorly. The distal condyles of the quadrate are situated well posteriorly from the level of the posterior margin of the occipital condyle, as also occurs in *Chanaresuchus bonapartei* (PULR 07, PVL 4586), *Gualosuchus reigi* (PVL 4576), *Tropidosuchus romeri* (PVL 4606), *Proterochampsa barrionuevoi* (PVSJ 77) and *Proterochampsa nodosa* (MCP 1694-PV). The distal condyles are taphonomically strongly dorsoventrally compressed in *Pseudochampsa ischigualastensis*, and the shape of their articular surfaces is obscured by the lower jaws.

#### Vomer

Most of the vomers (Figs. 2–4) is obscured by the fully occluded mandibular rami, but it can be observed that they contact the pterygoid and probably the palatine at their posterior end, as also occurs in *Chanaresuchus bonapartei* (PVL 4575). CT-scan images show that the vomers are broken at their anterior ends, which may be a consequence of the pressure supplied by the lower jaws during fossilization processes. The vomer of *Pseudochampsa ischigualastensis* is very narrow transversely, resembling the condition in *Chanaresuchus bonapartei* (PULR 07). By contrast, the vomer of *Proterochampsa barrionuevoi* seems to be proportionally broader (PVSJ 77). It is not possible to determine the presence of vomerine teeth in *Pseudochampsa ischigualastensis*.

#### Palatine

The palatine (Figs. 2–4) is mostly obscured by the lower jaws and the exposed surface is poorly preserved. The palatine forms the posterior border of the choana and the anterior border of the suborbital fenestra. The presence of teeth on the ventral surface of the palatine cannot be determined.

#### Pterygoids

The pterygoid (Figs. 2–4) is an anteroposteriorly very long bone that forms most of the palate. The pterygoids apparently contact with each other extensively along the median line of the palate, but it may be an artefact of post-mortem deformation because in other proterochampsids the pterygoids are separated from each other (e.g. *Chanaresuchus bonapartei*: PVL 4575; *Proterochampsa barrionuevoi*: PVSJ 77). Nevertheless, CT-scan images revealed that there is at least a long, slit-like interpterygoid vacuity approximately at the level of the palatines (Fig. 3B: iptv). The pterygoid possesses two distinct rows of palatal teeth (Fig. 4C: T2, T3). The first row ( = T3 of Welman [28]) is placed adjacent to the medial edge of the bone and extends anteriorly up to at least the level of the anterior border of the suborbital fenestra. The main axis of the second row ( = T2 of Welman [28]) is posteromedially-to-anterolaterally oriented, extending towards the palatine and may have extended onto this bone, as also occurs in *Proterosuchus fergusi* (RC 59) and *Proterochampsa barrionuevoi* (PVSJ 77). This second row of teeth is elevated from the rest of the surface of the pterygoid by a thick ridge, as also occurs in *Doswellia kaltenbachi* (USNM 214823) and other proterochampsids (e.g. *Chanaresuchus bonapartei*: PULR 07). Both rows of palatal teeth converge on the posteromedial end of the pterygoid, close to the basal articulation with the basipterygoid process. The lateral process of the pterygoid contacts the ectopterygoid, forming the pterygoid flange (sensu Romer [29]). The lateral process lacks palatal teeth, resembling the condition in other proterochampsids (e.g. *Chanaresuchus bonapartei*: PVL 4575; *Proterochampsa barrionuvoi* [23]). The quadrate ramus of the pterygoid is mostly broken, but the preserved portion shows that it is transversely compressed and posterolaterally oriented, resembling the condition in *Chanaresuchus bonapartei* (PULR 07). The quadrate ramus contacts the quadrate through an overlapping suture at the level of the basipterygoid processes.

#### Ectopterygoid

The ectopterygoid (Figs. 2–4) forms the posterior border of the suborbital fenestra. It contacts laterally the jugal and may have also contacted the maxilla. The ectopterygoid contacts medially the lateral process of the pterygoid, but the suture between both bones is not well preserved. The exposed overall morphology of the ectopterygoid is consistent with that of *Chanaresuchus bonapartei* (PULR 07) and *Proterochampsa barrionuevoi* (PVSJ 77).

#### Braincase

The braincase of *Pseudochampsa ischigualastensis* was described and figured in detail by Trotteyn & Haro [10]. We do not have additional information to provide here about this cranial region.

### Lower jaws

The lower jaws are fully occluded against the skull (Figs. 2–4) and, as a result, several features are obscured. Each dentary is bowed laterally along its extent and the anterior end of the bone is not transversely expanded. The symphysis between both dentaries is restricted to the anterior end of the bones. The CT-scan revealed at least eight tooth positions in the dentary and these alveoli are considerably smaller than those of the maxilla. The suture between the dentary, angular and surangular cannot be determined. The external mandibular fenestra cannot be identified on the lateral surface of the lower jaws, but it may be an artefact of the strong dorsoventral compression suffered by the skull. The lateral surface of the angular and probably surangular is invaded by an oval and moderately deep depression. There is no lamina on the ventral surface of the angular, contrasting with the condition in *Proterochampsa barrionuevoi* [23]. The retroarticular process is absent, resembling the condition in *Proterochampsa barrionuevoi* [23]. By contrast, the retroarticular process is very long in *Chanaresuchus bonapartei*, *Gualosuchus reigi* and *Tropidosuchus romeri* [6], [23].

### Postcranium

The postcranium of *Pseudochampsa ischigualastensis* is preserved mostly in articulation (Figs. 1, 5–13; Tables 1, 2), but suffered a strong dorsoventral post-mortem deformation. As a result, it is difficult to determine the detail anatomy of some regions, such as the pelvic girdle.

**Table 1:** Measurements of axial postcranial bones of *Pseudochampsa ischigualastensis* (PVSJ 567) in millimeters.

Element	Length	Width	Height
Third cervical centrum	17.3		
Anterior dorsal centrum	21.1		
Cervical rib	70.3		
Anterior dorsal neural spine	17.2		27.1
Second sacral centrum	22.6		
Anterior caudal centrum	24.3		
Chevron	44.4		
Osteoderm	10.4	4.4	

**Table 2:** Measurements of appendicular bones of *Pseudochampsa ischigualastensis* (PVSJ 567) in millimeters.

Element	Length	Maximum width
Proximal end of right humerus		37.8
Proximal end of left humerus		30.0
Humeral shaft		11.9
Right femur	140.2	16.7
Left femur	154.8	17.1
Right tibia	124.5	12.4
Left tibia	128.1	12.5
Right fibula	128.7	8.5
Left fibula	128.5	6.9
Right metatarsal I	17.0	5.2
Left metatarsal I	16.6	4.9
Right metatarsal II	46.1	9.8
Left metatarsal II	46.9	7.8
Right metatarsal III	53.4	8.1
Left metatarsal III	50.0	9.0
Right metatarsal IV	45.3	5.0
Left metatarsal IV	46.0	3.5
Right metatarsal V	13.6	4.5
Left metatarsal V	15.1	4.5

### Axial skeleton

The precaudal vertebral series of PVSJ 567 is complete, being composed of nine cervical (Figs. 1, 5, 6), 12 dorsal (Figs. 1, 7, 8) and two sacral (Fig. 8) vertebrae (Table 1). The first 18 caudal vertebrae are preserved (Figs. 9). The neck is proportionally short, representing approximately 0.35 times the length of the dorsal series (Fig. 1). The vertebrae are generally well-preserved but present some degree of port-mortem dorsoventral compression. The vertebrae are short, tall, amphicoelous and transversely compressed at the mid-length of the centrum, acquiring a spool shape in ventral view.

#### Atlas-axis complex

The proatlas is not preserved, but the atlantal neural arches and intercentrum are available (Figs. 5, 6). The right atlantal neural arch is the best preserved, but is overlapped laterally by the proximal end of the first cervical rib. Only the ventral end of the left neural arch is exposed. The atlantal neural arch is subtriangular in lateral view and the ventral end is transversely broader than the dorsal end. The dorsal end possesses a sharp edge that is narrower at its posterior half. The lateral surface is dorsoventrally convex and the medial surface is concave, as also occurs in *Chanaresuchus bonapartei* (PULR 07, PVL 4575). By contrast, the atlantal neural arch of *Tropidosuchus romeri* (PVL 4601, PVL 4602, PVL 4606) has a T-shape dorsal end, which is absent in *Chanaresuchus bonapartei* (PULR 07, PVL 4575) and *Pseudochampsa ischigualastensis*. The facet for reception of the occipital condyle is oval and situated anteroventrally. In the anteroventral portion of the bone there is also an anterodorsally facing narrow area that contacts the exoccipital. The intercentrum is a small, crescentic bone in anterior view that contacts the occipital condyle. This bone possesses two dorsolaterally oriented articular facets for articulation with its respective neural arches.

The centrum of the axis is longer than those of the postaxial cervical vertebrae and possesses a strong median ventral keel (Fig. 6). The ventral keel is extended along the entire surface of the centrum. The lateral surface of the centrum possesses a single, ventrally oriented rib facet, indicating that the axial rib was probably holocephalous. The prezygapophysis does not extend anteriorly beyond the level of the anterior margin of the centrum and the postzygapophysis possesses a transversely wide and posteroventrally facing articular facet. Only the posterior portion of the neural spine is preserved, but indicates that it should have been anteroposteriorly short. The posterior margin of the neural spine exceeds the level of the posterior margin of the postzygapophyses. The overall morphology of the atlas-axis complex of *Pseudochampsa ischigualastensis* is consistent with that of *Chanaresuchus bonapartei* (PULR 07).

#### Postaxial cervical vertebrae

The postaxial cervical series of *Pseudochampsa ischigualastensis* does not have intercentra (Figs. 5, 6), as also occurs in *Euparkeria* and more crownward archosauriforms [30]. The centra are subrectangular in lateral view, being longer than high, with both anterior and posterior articular surfaces situated at the same level. A well-developed ventral keel is present along the entire ventral surface of the cervical centra (Fig. 6), resembling the condition in *Proterochampsa barrionuevoi* (PVSJ 606) and *Chanaresuchus bonapartei* (PULR 07). The morphology of the parapophyses is obscured by the articulated cervical ribs (Figs. 5, 6). The lateral development of the diapophysis and size of its articular surface increase posteriorly in the cervical series. The same condition is observed in *Chanaresuchus bonapartei* (PULR 07) and *Proterochampsa barrionuevoi* (PVSJ 606), but in the latter species the articular surfaces are proportionally larger. The base of the diapophysis is situated in the centrum and its articular facet is sub-circular. The prezygapophysis has a transversely broad and dorsomedially facing articular surface, similar to those of *Chanaresuchus bonapartei* (PULR 07). The articular facets of the postzygapophyses are lateroventrally facing. Prezygapophyses and postzygapophyses are more divergent laterally from each other, respectively, than in *Chanaresuchus bonapartei* (PULR 07). Only the bases of the cervical neural spines are preserved and they are situated at the level of mid-length of the centrum (Fig. 5). By contrast, in *Chanaresuchus bonapartei* (PULR 07) the neural spines are situated at the level of the posterior half of the centrum. A deep post-spinal fossa is present on the base of the neural spine.

#### Dorsal vertebrae

The dorsal series also lack intercentra. The dorsal vertebral centra are sub-quadrangular in lateral view (Figs. 7, 8), and the anterior dorsal centra are axially shorter than those of the posterior dorsal ones. Because of the strong post-mortem compression it is not possible to determine if the centra were amphicoelous. The first and second dorsal vertebrae have a ventral median keel on their centrum, and this structure disappears in more posterior vertebrae, as also occurs in *Chanaresuchus bonapartei* (PULR 07, PVL 4575, PVL 6244) and *Proterochampsa barrionuevoi* (PVSJ 606). The lateral surface of the centra possesses a shallow lateral fossa. The neural arch lacks laminae, as also occurs in *Proterochampsa barrionuevoi* (PVSJ 606). By contrast, *Chanaresuchus bonapartei* possesses anterior and posterior centrodiapophyseal laminae on the dorsal vertebrae (MCZ 4037). Prezygapophyses are extended anteriorly at the same level of the anterior margin of the centrum (Fig. 8), contrasting with *Chanaresuchus bonapartei* (MCZ 4037, PULR 07, PVL 4575) and *Proterochampsa barrionuevoi* (PVSJ 606), in which the prezygapophysis is anteriorly extended beyond the level of the anterior articular surface of the centrum. The postzygapophysis extends posteriorly beyond the level of the posterior margin of the centrum (Fig. 8), resembling the condition in *Chanaresuchus bonapartei* (PULR 07, PVL 4575, 6244) and *Proterochampsa barrionuevoi* (PVSJ 606). The dorsal vertebrae of *Pseudochampsa ischigualastensis*, *Chanaresuchus bonapartei* (PULR 07, PVL 4575) and *Proterochampsa barrionuevoi* (PVSJ 606) lack hyposphene-hypantrum. The base of the neural arch possesses a strongly concave margin immediately ventral to the base of the prezygapophysis and there is a shallow groove separating both postzygapophyses from each other in the median line.

Only an anterior and a middle dorsal vertebra preserve an almost complete neural spine (Fig. 8). The anterior dorsal neural spine is sub-quadrangular in lateral view and lacks a spine table, resembling the condition in *Chanaresuchus bonapartei* (MCZ 4037, PULR 07, PVL 4575, 6244) and *Proterochampsa barrionuevoi* (PVSJ 606). The anteroposterior length of the base of this neural spine is similar to those of the cervical series. The neural spine of the middle dorsal vertebra is taller than long (Fig. 7), being rectangular in lateral view, resembling the condition in several basal archosauriforms (e.g. *Chanaresuchus bonapartei*: MCZ 4037, PVL 6244; *Erythrosuchus africanus*: NHMUK R3592). The distal end of the middle dorsal neural spine is not preserved. The middle and posterior dorsal neural spines are situated at the level of the posterior half of the centrum.

#### Sacral vertebrae

The pelvis is strongly dorsoventrally compressed by post-mortem deformation and severely damaged. As a result, it is very difficult to determine details of the sacrum (Fig. 8). The sacrum is composed of two vertebrae, as also occurs in several other basal archosauriforms (e.g. *Proterosuchus fergusi*: NM QR 1484; *Erythrosuchus africanus*: NHMUK R3592; *Euparkeria capensis*: SAM-PK-6048; *Chanaresuchus bonapartei*: PULR 07, MCZ 4035; *Proterochampsa barrionuevoi*: PVSJ 606). By contrast, *Doswellia kaltenbachi* possesses three sacral vertebrae [31].

#### Caudal vertebrae

The preserved caudal series of *Pseudochampsa ischigualastensis* is composed of 18 vertebrae that are articulated with the sacrum (Fig. 9). These vertebrae suffered strong post-mortem deformation, which consist of a dorsoventral compression more accentuated in the more posteriorly preserved caudals. No neural spine is completely preserved. The caudal series lacks intercentra. The centra lack a lateral fossa and are subrectangular in lateral view, being longer than tall. The centra become proportionally longer in the more posterior caudals, as also occurs in *Chanaresuchus bonapartei* (MCZ 4035, PVL 4575) and *Proterochampsa barrionuevoi* (PVSJ 606). The centra are compressed transversely at mid-length, being spool-shaped in ventral view (Fig. 9), resembling the condition in *Chanaresuchus bonapartei* (MCZ 4035, PVL 4575) and *Proterochampsa barrionuevoi* (PVSJ 606). The ventral surface of the centra possesses a pair of longitudinal ridges that starts on the posterior margin and reaches anteriorly the mid-length of the bone (Fig. 9). These ridges delimit a median groove that is shallow in anterior caudal vertebrae and become deeper posteriorly in the series. This groove is not present in *Chanaresuchus bonapartei* (MCZ 4035, PVL 4575) and *Proterochampsa barrionuevoi* (PVSJ 606). The articular surfaces of the centra are circular and the posterior one is more ventrally extended than the anterior one.

The prezygapophyses have a small articular surface through the caudal series, which is dorsally oriented in anterior caudal vertebrae and dorsomedially in more posterior vertebrae (Fig. 9). The prezygapophyses are well anteriorly extended, contrasting with the shorter prezygapophyses of *Chanaresuchus bonapartei* (MCZ 4035, PVL 4575) and *Proterochampsa barrionuevoi* (PVSJ 606). Middle caudal vertebrae have widely transversely separated prezygapophyses from each other, forming a U-shaped notch between them in dorsal view (Fig. 9). The postzygapophyses are also widely separated from each other in dorsal view. The wide transverse separation between each pair of apophyses is an autapomorphy of *Pseudochampsa ischigualastensis*, being absent in *Chanaresuchus bonapartei* (MCZ 4035, PVL 4575) and *Proterochampsa barrionuevoi* (PVSJ 606). The postzygapophyses are posteriorly projected up to the same level as the margin of the posterior articular surface of the centrum. The caudal series lacks hyposphene-hypantrum, as also occurs in *Chanaresuchus bonapartei* (MCZ 4035, PVL 4575) and *Proterochampsa barrionuevoi* (PVSJ 606). The transverse processes have an oval cross-section at their base and towards their distal end become more laminar (Fig. 9), resembling the condition in *Chanaresuchus bonapartei* (MCZ 4035, PVL 4575). The transverse processes possess a ventral, shallow longitudinal groove extended from the base to the distal end of the structure, which is absent in *Chanaresuchus bonapartei* (MCZ 4035, PVL 4575). The transverse processes decrease in width and length towards the posterior tip of the tail, being reduced to low protuberances in the tenth and eleventh caudal vertebrae.

#### Ribs

The holotype of *Pseudochampsa ischigualastensis* preserves most of the cervical ribs, lacking only their distal ends (Figs. 5, 6). The anterior cervical ribs are gracile, dichocephalous and have a posterior longitudinal groove extensively developed along the shaft, resembling the overall rib morphology present in *Chanaresuchus bonapartei* (MCZ 4038) and *Proterochampsa barrionuevoi* (PVSJ 606). The angle formed between the proximal end and the shaft is of approximately 175° and the shaft is posteroventrally oriented with respect to the dorsoventral axis of the proximal end. Posterior cervical ribs have the same morphology as the anterior cervical elements but are more robust and anteroposteriorly compressed. Dorsal ribs are similar to the posterior cervical ones, but the former have better developed tuberculi and capituli, and longer shafts (Figs. 5, 6). The morphology of the sacral ribs cannot be determined. Caudal ribs are fused to the transverse processes and form strongly laterally developed structures in the anterior region of the tail, resembling the condition in other proterochampsids (e.g. *Chanaresuchus bonapartei*: MCZ 4575) and doswelliids (e.g. *Doswellia kaltenbachi*: USNM 244214).

#### Haemal arches

Several haemal arches are preserved in articulation with their respective vertebra. The articular peduncles of the haemal arches diverge dorsolaterally from each other, resulting in a subtriangular haemal canal in anterior and posterior views (Fig. 9). The bases of the haemal blades are plate-like and subrectangular in lateral view in the anterior caudal series. By contrast, the haemal blades of middle caudal vertebrae taper slightly towards their distal end.

#### Gastralia

A portion of the gastral basket formed by series of small, elongated and rod-like bones is preserved in a separate block (Fig. 10). The diameter of the gastralia is smaller than that of the anterior cervical ribs, preventing their identification as the distal tips of the latter bones. The gastralia maintain the same anteroposterior depth along their width and are mostly parallel with each other, only contacting with each other at their distal ends. The number of segments in each row is unknown. The gastralia possess a Y-shaped expansion in the area that they articulate with its counterpart, resembling the condition in *Euparkeria capensis* (SAM-PK-5867) and *Proterochampsa barrionuevoi* (PVSJ 606), but this expansion is better developed in the latter species.

### Pectoral girdle

The pectoral girdle is represented by the right clavicle, scapula and coracoid, and the left clavicle and proximal end of the scapula, and three fragments of a possible interclavicle (Figs. 5, 6).

#### Interclavicle?

The largest preserved fragment of the interclavicle is placed between both clavicles. This fragment should belong to the posterior ramus of the interclavicle and is a flattened, rod-like bone slightly longer than the anteroposterior length of the coracoid (Fig. 6). The other two fragments are preserved next to the lateral surface of each clavicle. The main axis of the fragment that lies on the left clavicle is parallel to that of the clavicle. The fragment that lies on the right clavicle is perpendicular to the longitudinal axis of the vertebral series.

#### Clavicle

The clavicle (Fig. 6) is a slightly bowed, rod-like bone. It is difficult to determine the orientation of curvature of the clavicle. Both clavicles are anteromedially-to-posterolaterally oriented as preserved, with their distal ends directed towards the acromion process of the scapulacoracoids. The distal ends of the clavicles are covered by the scapulacoracoid on the right side and matrix on the left side. The overall morphology of the clavicle resembles that of other basal archosauriforms (e.g. *Garjainia prima*: PIN 2394/5-35).

#### Scapulacoracoid

The scapula and coracoid are fused with each other without visible line of suture (Figs. 5, 6), as also occurs in *Proterochampsa barrionuevoi* (PVSJ 606). By contrast, in preserved specimens of *Chanaresuchus bonapartei* (MCZ 4035, PVL 4575) the scapula and coracoid are not fused with each other. The scapular blade is tall and anteroposteriorly narrow (Figs. 5, 6), and the distal end is less expanded than in *Chanaresuchus bonapartei* (MCZ 4035, PVL 4575) and possibly *Proterochampsa barrionuevoi* (PVSJ 606). The contact between the scapula and coracoid does not have a notch on its anterior margin (Fig. 6), resembling the condition in *Chanaresuchus bonapartei* (MCZ 4035, PVL 4575). The scapular neck has an oval, small tubercle adjacent to its posterior margin, which may corresponds to the insertion of the triceps muscle. The proximal end of the scapula is moderately anteroposteriorly expanded. The supraglenoid lip is well-developed and posterolaterally oriented, as also occurs in other proterochampsids [19]. The glenoid fossa is posteroventrally facing. Because of the poor state of preservation of the anteroventral edge of the scapula the region of contact with the clavicle could not be identified.

Only the ventral surface of the coracoid is exposed because the dorsal half of the bone is overlapped by a series of cervical ribs (Fig. 6). The coracoid is oval, being anteroposteriorly longer than tall, resembling the condition in *Chanaresuchus bonapartei* (PVL 4575). The ventral margin is strongly anteroposteriorly convex. The anterior margin of the coracoid is rounded and thin, while the posterior edge is slightly thicker. The coracoid foramen is small and placed anteroventrally to the glenoid fossa (Fig. 6).There is no distinct posteroventral (or sternal) process on the posterior margin of the coracoid.

### Forelimb

#### Humerus

The proximal halves of both humeri (exposed in ventral view) are preserved (Figs. 5, 6; Table 2). The proximal end of the humerus is strongly transversely expanded, resembling the condition in *Proterochampsa barrionuevoi* (PVSJ 606), but contrasting with the proportionally narrower proximal end of *Chanaresuchus bonapartei* (MCZ 4035, PULR 07, PVL 4575, 6244). The proximal end is anteroposteriorly compressed (Fig. 6) and this condition is not a result of post-mortem compression because the deltopectoral crest is not deformed. The anterolateral surface of the proximal end possesses a low deltopectoral crest, which is oriented parallel to the main axis of the bone and reaches the proximal portion of the shaft. The morphology of the deltopectoral crest is similar to that of *Chanaresuchus bonapartei* (MCZ 4035, PULR 07, PVL 4575) and *Proterochampsa barrionuevoi* (PVSJ 606). However, in *Proterochampsa barrionuevoi* the deltopectoral crest is less rounded in lateral view (PVSJ 606). The anterior surface of the shaft possesses a triangular, concave depression, which is delimited laterally by the deltopectoral crest and finishes distally at the same level as the distal end of the crest. This depression is also present in *Chanaresuchus bonapartei* (PULR 07, MCZ 4035, PVL 4575) and *Tropidosuchus romeri* (PVL 4601, 4602, 4606), but is absent in *Proterochampsa barrionuevoi* (PVSJ 606). The preserved portion of shaft is oval in cross section, being transversely broader than anteroposteriorly deep. The presence of a twisted humeral shaft cannot be determined because the distal half of the bone is missing.

### Pelvic girdle

The pelvic girdle is the region of the skeleton that suffered more dorsoventral compression (Figs. 8, 12). The pelvic girdle is propubic. The acetabulum is composed of the three pelvic bones and seems to be fully closed in medial view. The lateral surface of the acetabulum is overlapped by the femoral head on both sides.

#### Ilium

The iliac blade is dorsoventrally low above the acetabulum and transversely compressed. The preacetabular process is short and does not extend beyond the anterior margin of the acetabulum, resembling the condition in other proterochampsids (e.g. *Chanaresuchus bonapartei*: MCZ 4035). The postacetabular process is well posteriorly extended and possesses a tapering posterior end, resembling the condition in *Chanaresuchus bonapartei* (MCZ 4035, PVL 4575). By contrast, the posterior end of the postacetabular process is more rounded in *Proterochampsa barrionuevoi* (PVSJ 606). The postacetabular process of *Pseudochampsa ischigualastensis* possesses a laterally facing depression that extends from the posterior margin of the acetabulum to the posterior tip of the iliac blade, becoming shallower posteriorly. This depression is not present in *Chanaresuchus bonapartei* (MCZ 4035, PVL 4575, 6244). The medial surface of the ilium is severely damaged, but shallow depressions for the possible articulation of sacral ribs are discerned.

#### Pubis

Both pubes are preserved (Figs. 8, 12), but the puboischiadic plate and the symphyseal region are not completely preserved. The iliac peduncle is small and anterolaterally placed in the proximal end of the bone. The articulation between the pubis and ilium is anteroposteriorly broad. The presence of an obturator foramen cannot be determined because of severe breakage. The pubic shaft is rod-like and curves slightly posteriorly, as also occur in *Chanaresuchus bonapartei* (MCZ 4035, PVL 4575). The pubic apron is transversely very broad, resembling the condition in other basal archosauriforms (e.g. *Chanaresuchus bonapartei*: PVL 4575).

#### Ischium

Only the acetabular region of the bone seems to be preserved and is not very informative.

### Hindlimb

Femora, tibiae and fibulae are fairly complete, but their proximal and distal ends are strongly taphonomically compressed (Figs. 8, 11, 12; Table 2).

#### Femur

The femur is sigmoid in anterior view (Figs. 11, 12), resembling the condition in other proterochampsids (e.g. *Chanaresuchus bonapartei*: PVL 4575; *Gualosuchus reigi*: PVL 4576; *Proterochampsa barrionuevoi*: PVSJ 606; *Tropidosuchus romeri*: PVL 4601, PVL 4602). Both ends are anteroposteriorly expanded and the shaft is oval in cross-section, with an anterolaterally oriented main axis. The femoral head is rounded in posterior view and is not separated from the shaft by a distinct neck, contrasting with the condition in dinosauriforms [32], [33]. The angle formed between the femoral head and the shaft is of around 30° in posterior view, resembling the condition in *Chanaresuchus bonapartei* (PULR 07, MCZ 4035, PVL 4575, 6244), but contrasting with the 45° present in *Proterochampsa barrionuevoi* (PVSJ 606). The femoral head possesses a shallow and transversely broad fossa on its anterior surface, which extends onto the proximal two thirds of the bone (Figs. 11, 12). The same condition is present in *Chanaresuchus bonapartei* (MCZ 4035, PULR 07, PVL 4575) and *Proterochampsa barrionuevoi* (PVSJ 606). The femur lacks an anterior trochanter. The fourth trochanter is placed on the proximal third of the bone and its main axis is parallel to the shaft (Fig. 12: ft). By contrast, in *Chanaresuchus bonapartei* (MCZ 4035, PULR 07, PVL 4575, 6244) and *Proterochampsa barrionuevoi* (PVSJ 606) the fourth trochanter extends onto the middle third of the bone. The fourth trochanter of *Pseudochampsa ischigualastensis* is posterolaterally oriented and subtriangular in medial view. The degree of posterior development of the fourth trochanter of *Pseudochampsa ischigualastensis* is similar to that of *Chanaresuchus bonapartei* (MCZ 4035, PULR 07, PVL 4575), but contrasts with the smaller structure of *Proterochampsa barrionuevoi* (PVSJ 606). A shallow groove with a Y-shaped distal end is present immediately distally to the fourth trochanter, which is identified as the adductor ridge (sensu Romer [29]). The distal end is strongly compressed by post-mortem deformation and, as a result, the popliteal and extensor fossae cannot be distinguished. The medial surface of the distal end possesses a concave depression that extends along the distal two-thirds of the bone.

#### Tibia

The tibia represents 82.7–88.8% of the length of the femur (Figs. 1, 11, 12). The proximal and distal ends are anteroposteriorly expanded and there is a slight torsion between their main axes. The proximal end of the bone is overlapped by the femur and strongly taphonomically compressed. As a result, the proximal condyles cannot be clearly distinguished. However, a low cnemial crest is exposed on the proximal end of the left tibia, resembling the condition in *Chanaresuchus bonapartei* (MCZ 4035, PVL 4575) and *Proterochampsa barrionuevoi* (PVSJ 606). The shaft is oval in cross section, with an anteroposterior main axis. The lateral surface of the distal end has a shallow longitudinal groove that reaches the distal articular surface of the bone (Fig. 11: lg). The medial surface of the distal end possesses a subtriangular depression, with a proximally oriented apex, that extends along the distal quarter of the bone. The distal articular surface is damaged and covered by proximal tarsals, and as a result, its detailed morphology cannot be described.

#### Fibula

The fibula is slightly narrower than the tibia (Figs. 1, 11, 12), resembling the condition in *Chanaresuchus bonapartei* (MCZ 4035, PVL 4575) and *Proterochampsa barrionuevoi* (PVSJ 606). Both ends are expanded, but the main axis of the proximal end is twisted approximately 85° with respect to the distal end, resembling the condition in other proterochampsids [19]. As a result, the proximal end is mainly transversely expanded and the distal end is mainly anteroposteriorly expanded. The shaft has an oval cross section, being anteroposteriorly deeper than transversely broad, and decreases its diameter towards the proximal end. The tubercle for insertion of the *M. iliofibularis* is absent, as in other proterochampsids [19], [31].

#### Astragalus and calcaneum

The proximal tarsals are strongly compressed and rather damaged (Figs. 8, 11, 12). The contact between the proximal tarsals consists of two articular surfaces (sensu Arcucci [34]). The first one is represented by a rounded process on the astragalus that faces ventrolaterally and articulates on the medial surface of the calcaneum. The second articulation is formed by a medially expanded posterior projection on the calcaneum that articulates with the posterolateral surface of the astragalus. The dorsal surface of the astragalus is divided into distinct articular facets for the tibia and fibula, respectively. The tibial facet is dorsally facing and covers most of the dorsal surface of the bone, as it is the case in *Chanaresuchus bonapartei* (MCZ 4035) and *Proterochampsa barrionuevoi* (PVSJ 606). This facet is sub-quadrangular in medial view and has a laterally oriented crest that divides it into two differently oriented surfaces, conferring a saddle-shape to the astragalus in dorsal view. The tibial facet of the astragalus is not completely exposed, but exposed region is concave in anterior view and the astragalocalcaneal articular surface is flat. The fibular facet is smaller than the tibial one and dorsally facing, contrasting with the dorsolaterally oriented fibular facets of *Chanaresuchus bonapartei* (MCZ 4035) and *Proterochampsa barrionuevoi* (PVSJ 606). The fibular facet of *Pseudochampsa ischigualastensis* is quadrangular and surrounded anteriorly by a low rim. As in other proterochampsids [30], the astragalocalcaneal ventral articular facet is small. The anterior surface of the astragalus has a concave surface that may be homologous to the anterior hollow [30], [35]. The astragalus possesses a posterior groove (sensu Sereno [30]) that is delimited by two ridges and is transversely developed in posterior view. The surface of the posterior groove apparently lacks the foramina present in *Chanaresuchus bonapartei* (MCZ 4035), but the condition in *Pseudochampsa ischigualastensisi* may be a post-mortem artefact. The calcaneum is taphonomically compressed and broken, but it can be discerned that is transversely narrower than the astragalus. The calcaneal tuber is ventrally displaced from the rest of the calcaneum by taphonomic processes, but it should have been posterolaterally or laterally oriented in life. The calcaneal tuber is rectangular, being taller than wide. The distal surface of the tuber is flat and the medial one is concave. The medial surface of the calcaneum articulates with the astragalus and the dorsal surface on its medial half receives the fibula.

#### Distal tarsals

The proximal tarsals and metatarsals articulate with a series of poorly preserved, small bones that should correspond topologically to the distal tarsals. These bones are poorly preserved and not very informative.

#### Foot

Both feet are almost complete, lacking only some phalanges (Figs. 8, 11, 12). The metatarsals overlap each other at their proximal ends and diverge from the ankle. The proximal end of the metatarsal I cannot be observed because is overlapped by the metatarsal II. The distal end of the metatarsal I is expanded laterally. Digit I is preserved in natural articulation. The phalanx I-1 has collateral ligament facets and a ginglymoid distal end. The ungual of the first digit has a groove on its lateral surface that is extended from the proximal end to the two distal third of the bone. This ungual is ventrally recurved, as also occurs in the other pedal ungual phalanges of *Pseudochampsa ischigualastensis*.

Metatarsal II is the most robust element of the metatarsus. This metatarsal has transversely expanded ends and possesses a shallow, longitudinal groove that extends onto both ventral and dorsal surfaces of the shaft, as also occurs in metatarsal III. The second digit is composed of three phalanges, in which the non-ungual phalanges have strongly developed distal ginglymoid facets and deep collateral ligament fossae. Phalanx II-1 is the longest and widest of the series. The ungual phalanx of the second digit is longer than that of the first digit.

Metatarsal III is the longest of the metatarsus and has transversely expanded ends. The third digit is composed of four phalanges, in which phalanx III-1 is the longest. All the phalanges have strongly developed distal ginglymoid facets and shallow collateral ligament fossae. The ungual phalanx has the same length as that of the second digit.

Metatarsal IV is the most gracile element of the metatarsus. This metatarsal lacks conspicuous expanded ends. The fourth digit is completely missing. Metatarsal V is a small, ventrally curved bone that lacks a distal articular surface. This metatarsal is slightly longer than phalanx I-1. The overall morphology of the foot of *Pseudochampsa ischigualastensis* resembles the apomorphic condition observed in other proterochampsids, including the presence of a robust metatarsal II and a gracile metatarsal IV.

#### Osteoderms

The holotype of *Pseudochampsa ischigualastensis* has several well-preserved paramedian osteoderms associated with the dorsal and sacral vertebrae (Figs. 3, 7, 13). The osteoderms are not in their original position, but preserved on the lateral surfaces of the vertebrae and pelvic girdle. It is interpreted that *Pseudochampsa ischigualastensis* had a single row of paramedian osteoderms on the dorsal and sacral vertebral series. Although it seems that osteoderms were absent on the cervical and caudal regions, the condition cannot be determined with confidence. By contrast, in *Chanaresuchus bonapartei* the osteoderms are present from the second cervical to the last dorsal vertebra (PULR 07, MCZ 4037, PVL 4575, 6244) and they are completely absent in *Proterochampsa barrionuvoi* [23]. The osteoderms of *Pseudochampsa ischigulastensis* are X-shaped in dorsal view, with concave margins in dorsal view and an anteroposteriorly oriented main axis (Fig. 13). By contrast, *Chanaresuchus bonapartei* has wedge-shaped osteoderms (PULR 07, MCZ 4037, PVL 4575, 6244) and *Tropidosuchus romeri* possesses heart-shape osteoderms (PVL 4601, 4602). The dorsal surface of the osteoderms of *Pseudochampsa ischigualastensis* possesses a deep, well-defined longitudinal groove that covers the entire length of the element. This feature is absent in *Chanaresuchus bonapartei* (PULR 07, MCZ 4037, PVL 4575, 6244) and *Tropidosuchus romeri* (PVL 4601, 4602).

## Discussion

### Phylogenetic analysis

**Figure 14:**
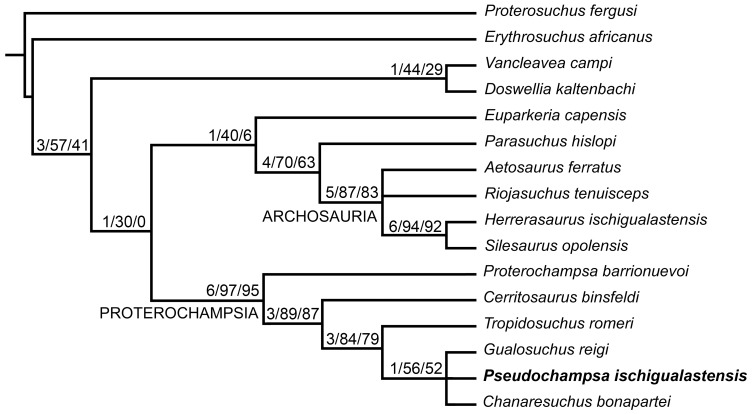
Phylogenetic relationships of *Pseudochampsa ischigualastensis* and other proterochampsians among basal archosauriforms. Numbers above nodes are Bremer support, absolute and GC bootstrap frequencies, respectively.

The search recovered two most parsimonious trees of 200 steps, with a consistency index of 0.5950 and a retention index of 0.7128. The overall topology of the strict consensus tree (Fig. 14) is consistent with that originally obtained by Dilkes & Arcucci [23], but it differs in the lack of resolution at the base of Archosauria, in which is found a trichotomy composed of *Aetosaurus ferratus*, *Riojasuchus tenuisceps* and Avemetatarsalia. The lack of resolution at this node is very likely result of poor character and taxonomic sampling for that part of the tree. Proterochampsia is recovered as a monophyletic group and *Pseudochampsa ischigualastensis* is found within this clade, in agreement with previous qualitative analyses [9]. The monophyly of Proterochampsia is supported by 12 synapomorphies (characters 1, 7, 9–11, 15, 20, 26, 39, 42–44) that were already discussed in detail by Dilkes & Arcucci [23] (see Appendix S3). Proterochampsia has a decay index of 6 and absolute and GC bootstrap frequencies of 97% and 95%, respectively. The clade composed of *Cerritosaurus binsfeldi*, *Tropidosuchus romeri*, *Chanaresuchus bonapartei*, *Gualosuchus reigi* and *Pseudochampsa ischigualastensis* has three synapomorphies (characters 8, 23 and 24) that were also already discussed by Dilkes & Arcucci [23] (see Appendix S3). The decay index of this clade is 3 and absolute and GC bootstrap frequencies are 89% and 87%, respectively. The node that includes *Tropidosuchus romeri*, *Chanaresuchus bonapartei*, *Gualosuchus reigi* and *Pseudochampsa ischigualastensi* has three synapomorphies (characters 19, 45 and 46) that were already discussed by Dilkes & Arcucci [23] (see Appendix S3). The decay index of the node is 3 and the absolute and GC bootstrap frequencies of 84% and 79%, respectively.

The less inclusive clade of Proterochampsia is represented by a trichotomy composed of *Gualosuchus reigi*, *Chanaresuchus bonapartei* and *Pseudochampsa ischigualastensis*. This clade is diagnosed by a skull with a box-like rostrum anterior to the prefrontal, formed by a sharp inflexion between the lateral and dorsal surfaces of the maxilla (character 11), and dorsal surface of nasals and/or frontals ornamented by ridges radiating from centres of growth (character 106) (see Appendix S3). This node has a decay index of 1 and absolute and GC frequencies of 56% and 52%, respectively.

Under suboptimal constrained topologies two additional steps (TL = 202 steps) are necessary to recover *Pseudochampsa ischigualastensis* as the sister taxon of *Tropidosuchus romeri*, four additional steps (TL = 204 steps) to force it as the sister-taxon of *Cerritosaurus binsfeldi*, and seven additional steps (TL = 226 steps) to force it as the sister-taxon of *Proterochampsa barrionuevoi*. In summary, *Pseudochampsa ischigualastensis* is found here as a derived member of Proterochampsia and Proterochampsidae, enclosed within a group also composed of *Chanaresuchus bonapartei* and *Gualosuchus reigi*.

### Taxonomy of ‘*Chanaresuchus*’ *ischigualastensis*

Trotteyn et al. [9] erected the new proterochampsid genus and species ‘*Chanaresuchus*’ *ischigualastensis*. These authors assigned the new species to the genus *Chanaresuchus* based on the presence of a skull ornamented by longitudinal crests and depressions on the dorsal surfaces of the premaxillae, maxillae, and nasals; presacral vertebrae with a lateral fossa on the centrum; humerus with low deltopectoral crest; and absence of pedal digit V (Trotteyn et al. [9]: p. 488). Subsequently, the latter taxonomic hypothesis was followed by Trotteyn et al. [6]. In the present phylogenetic analysis we did not find unambiguous evidence to support the monophyly of *Chanaresuchus* because of the presence of a trichotomy composed of ‘*Chanaresuchus*’ *ischigualastensis*, *Chanaresuchus bonapartei* and *Gualosuchus reigi*. In other words, the recovery of ‘*Chanaresuchus*’ *ischigualastensis* as the sister-taxon of *Gualosuchus reigi* is as parsimonious as the recovery of the former species as the sister-taxon of *Chanaresuchus bonapartei*. The latter result forces us to revisit the taxonomy of ‘*Chanaresuchus*’ *ischigualastensis*. The supposed synapomorphies that supported the assignment of this species to the genus *Chanaresuchus* deserve the following comments.

*Skull ornamented by longitudinal crests and depressions on the dorsal surfaces of the premaxillae, maxillae, and nasals*. Longitudinal ridges ornamenting dermal skull bones, mainly on the skull roof, are also present in *Gualosuchus reigi* (PULR 05, PVL 4576), *Chanaresuchus bonapartei* (PULR 07, MCZ 4037, 4039), *Rhadinosuchus gracilis* (BSPG AS XXV 50, 51) and ‘*Chanaresuchus*’ *ischigualastensis*. As a result, this character is not restricted to the genus *Chanaresuchus*.

*Presacral vertebrae with a lateral fossa on the centrum.* The lateral surface of the presacral centra of ‘*Chanaresuchus*’ *ischigualastensis* possesses a shallow, not well-rimmed depression, resembling the condition in *Chanaresuchus bonapartei* (PULR 07, MCZ 4037, PVL 6244), *Gualosuchus reigi* (PULR 05), *Rhadinosuchus gracilis* (BSPG AS XXV 50, 51) and *Tropidosuchus romeri* (PVL 4601).

*Humerus with low deltopectoral crest.* The humeri of ‘*Chanaresuchus*’ *ischigualastensis*, *Chanaresuchus bonapartei* (MCZ 4035), *Gualosuchus reigi* (PVL 4576) and *Tropidosuchus romeri* (PVL 4601) possess a low deltopectoral crest, contrasting with the condition in *Proterochampsa barrionuevoi* [27]. The condition of this character is unknown in *Rhadinosuchus gracilis*.

*Absence of pedal digit V.* ‘*Chanaresuchus*’ *ischigualastensis*, *Chanaresuchus bonapartei* [36] and *Tropidosuchus romeri* (PVL 4601) lack a pedal digit V, showing that this condition is widespread beyond the genus *Chanaresuchus* among proterochampsids.

In addition, Dilkes and Arcucci [23] proposed three autapomorphies for their diagnosis of *Chanaresuchus bonapartei*, namely premaxillae with midline posterior processes ( = prenarial process) longer than the posterodorsal processes ( = postnarial process), deep depression anterior to external naris with well-defined borders, and elongate oval supratemporal fenestrae with a posterolaterally directed main axis. The first character-state cannot be determined in ‘*Chanaresuchus*’ *ischigualastensis* because the contact between the prenarial process of the premaxillae and nasals cannot be discerned. The other two characters-states are absent in ‘*Chanaresuchus*’ *ischigualastensis*, in which the depression anterior to the external naris is shallow, resembling the condition in *Gualosuchus reigi* (PULR 05), and the supratemporal fenestra is subtriangular and aligned with the long axis of the skull. As a result, we could not find evidence for the monophyly of *Chanaresuchus* sensu Trotteyn et al. [9] in the diagnosis of *Chanaresuchus bonapartei* proposed by Dilkes and Arcucci [23].

Accordingly, we cannot sustain any synapomorphy or unique combination of apomorphies that may support the monophyly of the genus *Chanaresuchus*. In addition, the evidence currently available at hand may support, for example, the position of *Rhadinosuchus gracilis* as the sister-taxon of *Chanaresuchus bonapartei* rather than of ‘*Chanaresuchus*’ *ischigualastensis*. Accordingly, we propose here the erection of the new genus *Pseudochampsa* for ‘*Chanaresuchus*’ *ischigualastensis*, resulting in the new combination *Pseudochampsa ischigualastensis*.

The biogeographic and macroevolutionary history of proterochampsids is mostly ignored [6], [37] and the information provided here about the anatomy and taxonomy of *Pseudochampsa ischiguaslastensis* will be useful for future quantitative analyses focused on these topics.

## Supporting Information

Appendix S1 **Characters added to the data matrix of Dilkes and Arcucci** [23] **.**
(DOC)

Appendix S2 **Data matrix used in the present study.**
(DOC)

Appendix S3 **Synapomorphies common to all the recovered MPTs of nodes.**
(DOC)
