# Supplementary material for: Osteology of Pseudochampsa ischigualastensis gen. et comb. nov. (Archosauriformes: Proterochampsidae) from the Early Late Triassic Ischigualasto Formation of Northwestern Argentina
Source: PLoS One. 2014 Nov 26;9(11):e111388. doi: 10.1371/journal.pone.0111388 (PMC4245112; doi:10.1371/journal.pone.0111388)
Supplement: Appendix S1 — Characters added to the data matrix of Dilkes and Arcucci [23] . (DOC) [file pone.0111388.s001.doc]

**Supplementary information for Trotteyn and Ezcurra “Osteology of *Pseudochampsa ischigualastensis* gen. et comb. nov. (Archosauriformes: Proterochampsidae) from the early Late Triassic Ischigualasto Formation of northwestern Argentina”**

APPENDIX S1

Characters added to the data matrix of Dilkes and Arcucci [23]. The enumeration of character follows that of the original data set:

104. Skull, strongly dorsoventrally compressed skull with dorsally facing antorbital fenestrae and mainly dorsally facing orbits: absent (0); present (1) (taken from Reig [39])

105. Skull, well developed nodular prominences on the lateral surface of maxilla, jugal, quadratojugal, squamosal and angular: absent (0); present (1) (taken from Sill [18])

106. Skull, dorsal surface of nasals and/or frontals ornamented by ridges radiating from centres of growth: absent (0); present (1) (taken from Romer [28]). This character is inapplicable to taxa that lack ridges or tubercles on the dorsal surface of the skull roof (character 1: state 0).

107. Skull, supratemporal fossa: absent (0); present (1) (taken from Dilkes & Arcucci [23]). The character is inapplicable in taxa lacking a supratemporal fenestra (e.g. *Vancleavea campi*).

108. Lacrimal, antorbital fossa on ventral process: absent or strongly restricted anteriorly (0); occupies almost half or more of the anteroposterior length of the process (1). (New character) The character is inapplicable in taxa lacking an antorbital fenestra (e.g. *Vancleavea campi*).

109. Lower jaw, retroarticular process: absent or incipient (0); distinctly developed (1) (taken from Dilkes & Arcucci [23])

110. Angular, distinctly ventrally developed thick, longitudinal lamina on the ventral surface between the levels of the external mandibular fenestra and the mandibular glenoid fossa: absent (0); present (1) (taken from Dilkes & Arcucci [23])
