# Supplementary material for: Osteology of Pseudochampsa ischigualastensis gen. et comb. nov. (Archosauriformes: Proterochampsidae) from the Early Late Triassic Ischigualasto Formation of Northwestern Argentina
Source: PLoS One. 2014 Nov 26;9(11):e111388. doi: 10.1371/journal.pone.0111388 (PMC4245112; doi:10.1371/journal.pone.0111388)
Supplement: Appendix S2 — Data matrix used in the present study. (DOC) [file pone.0111388.s002.doc]

**Supplementary information for Trotteyn and Ezcurra “Osteology of *Pseudochampsa ischigualastensis* gen. et comb. nov. (Archosauriformes: Proterochampsidae) from the early Late Triassic Ischigualasto Formation of northwestern Argentina”**

APPENDIX S2

Data matrix used in the present study in TNT file:

xread

110 16

Proterosuchus_fergusi 00001000001001100000000000000?00000100000000000110?000100000000000000000??000000000000000000000000000??00-1110

Euparkeria_capensis 0100000000010100011010000010101110000001001000011001111?0001001010011011011000001110001011000000010010000-1010

Doswellia_kaltenbachi 0?????????????01110?0???0?0011102?01??01000000?001010100011011?????10001010?1???????????????????????10010-0?10

Erythrosuchus_africanus 01000000000?010001000000001011110001?1211?10000010000010011???1001011000010000001?00000011000000010????00-1010

Parasuchus_hislopi ?1000100000101001110000000011011210011210?00?01001011110111111100001101101?010001111101111000000010010000-1010

Proterochampsa_barrionuevoi 11000110111?011111010200011010?1210100110121001001?1001?01??????????????????????????????????????????0?01100001

Tropidosuchus_romeri 11000111111?001?1111111111?0???11?0??111112?11???11100110?1??0101??1?01101101010111000001100001101111100001010

Gualosuchus_reigi 11000111112??11111111111111?1???1?010011112111100?0?0?1???????????????1?????10??????0???????????????11?0011010

Chanaresuchus_bonapartei 11000111112?01111111111111101?11100100111121111001010011011???10100110110110101011?00000110000110111110[01]011110

Cerritosaurus_binsfeldi 110001111?1???111101?111?1??????????????????00100??????????????????????????????????????????????????????0001110

Vancleavea_campi 00???100??0?00011100010000?????????1??????0000???1110000?1??????1000????0100100010000000110?00??0???1?100---10

Riojasuchus_tenuisceps 01111000000?11001110100001121??1211111210?100011110?1?1????????1111???1?111111011121111111000000110?10000-1010

Aetosaurus_ferratus 01110000000??10011101001001200?12?11?1211?01000001????????11?01101111010111011011???1?1111000000110?10100-1110

Silesaurus_opolensis 01?000????0?110?111?1?0?00?01011210???210?01001?01010010011???11111111111211101011?120??11?1110011110??00-??10

Herrerasaurus_ischigualastensis 01100000000??10111101101000210??211111210?01001011?10110??1?????101?11111011101011?12???1111110011010??00-1110

Pseudochampsa_ischigualastensis 11000111112??1111111121111101?111011??11112111100101001101?0?010???11?1?0?10?010110?0000?1??00111?011101011000

;

ccode+21;

p/;
