# Supplementary material for: Osteology of Pseudochampsa ischigualastensis gen. et comb. nov. (Archosauriformes: Proterochampsidae) from the Early Late Triassic Ischigualasto Formation of Northwestern Argentina
Source: PLoS One. 2014 Nov 26;9(11):e111388. doi: 10.1371/journal.pone.0111388 (PMC4245112; doi:10.1371/journal.pone.0111388)
Supplement: Appendix S3 — Synapomorphies common to all the recovered MPTs of nodes. (DOC) [file pone.0111388.s003.doc]

**Supplementary information for Trotteyn and Ezcurra “Osteology of *Pseudochampsa ischigualastensis* gen. et comb. nov. (Archosauriformes: Proterochampsidae) from the early Late Triassic Ischigualasto Formation of northwestern Argentina”**

APPENDIX S3

Synapomorphies common to all the recovered MPTs of nodes that include *Pseudochampsa ischigualastensis*:

*Doswellia +* crownwards archosauriforms: 17(0→1), 33(0→2), 49(1→0), 50(0→1), 52(0→1), 65(0→1), 72(0→1), 77(0→1).

*Vancleavea + Doswellia*: 55(1→0).

Proterochampsia + cronwards archosauriforms: 30(1→0), 71(0→1), 75(0→1), 82(0→1).

Proterochampsia: 1(0→1), 7(0→1), 9(0→1), 10(0→1), 11(0→1), 15(0→1), 20(0→1), 26(0→1), 39(0→1), 42(0→1), 43(0→2), 44(0→1).

*Cerritosaurus + Chanaresuchus*: 8(0→1), 23(0→1), 24(0→1).

*Tropidosuchus* + *Chanaresuchus*: 19(0→1), 45(0→1), 46(0→1).

*Gualosuchus + Chanaresuchus + Pseudochampsa*: 11(1→2), 106(0→1).
